# Microglia Mitochondria Support Neuronal Maturation via Metabolic and Transcriptional Reprogramming in Human 3D In Vitro Brain Model

**DOI:** 10.1002/advs.202508815

**Published:** 2026-03-13

**Authors:** Sydney P. Sterben, Charitha C. Anamala, Sahan B. S. Kansakar, Vaishnavi Koduri, Volha Liaudanskaya

**Affiliations:** ^1^ Department of Biomedical Engineering University of Cincinnati Cincinnati Ohio USA; ^2^ Neuroscience Graduate Program College of Medicine University of Cincinnati Cincinnati Ohio USA

**Keywords:** autism spectrum disorder, microglia, mitochondria, neurodevelopment, neurodevelopment, transcriptional reprogramming

## Abstract

Autism Spectrum Disorder (ASD) is a neurodevelopmental condition characterized by disrupted neuronal circuit maturation. Emerging evidence implicates microglial function and mitochondrial regulation as contributors to ASD‐associated biology, yet the mechanisms linking these processes to neuronal development remain poorly defined. Neuronal maturation requires tightly coordinated metabolic and transcriptional remodeling, in which mitochondria play a central role in regulating the developmental tempo and metabolic identity, while microglia modulate neuronal synaptic network maturation; however, whether microglia influence neuronal development through direct mitochondrial contributions remains unknown. Here, using a 3D human in vitro brain model, it is shown that microglial mitochondria can act as transferable cues that promote metabolic, mitochondria‐dynamic, and transcriptional aspects of neuronal maturation. Neurons treated with microglial mitochondria exhibited enhanced oxidative metabolism, improved mitochondrial dynamics, and activation of gene programs associated with nervous system development and neurogenesis. These effects are accompanied by increased expression of dendritic maturation markers, supporting the view that transferred mitochondria can contribute to the regulation of neuronal state. However, full structural and synaptic maturation required the combined action of microglia‐derived mitochondria and secreted signaling factors. Together, this study identified microglial mitochondrial transfer as a contributor to neuronal maturation with potential relevance to developmental trajectories disrupted in ASD.

## Graphical abstract

xx xx.

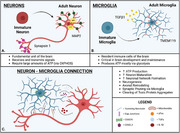

## 1 Introduction

The development and maturation of neurons is a well‐orchestrated and highly dynamic process that guides functional neural circuit formation [1]. This process extends from early neurogenesis through adolescence and adulthood, particularly in the human brain, which undergoes significant alterations after birth. Neuronal maturation is regulated by a complex interplay of intrinsic genetic programs and extrinsic environmental signals, including neuron‐glia interactions, synaptic activity, secreted molecules, metabolic state, and mitochondrial function [2, 3]. Any disruption in these developmental programs can lead to broad alterations in neuronal architecture and function, manifesting in abnormal synapse formation, delayed neurite outgrowth, impaired GABAergic signaling, increased oxidative stress, and neuroinflammation [4].

Autism spectrum disorder (ASD) is a neurodevelopmental condition increasingly associated with disrupted neuronal maturation [5]. Affecting approximately 1 in 36 children in the United States [6], ASD is characterized by deficits in social interaction and communication, along with restrictive and repetitive behaviors [7]. While genetics plays a large role in the development of ASD, it is important to acknowledge that environmental and immunological factors play a critical role in ASD [8]. This includes the use of certain pharmaceutical drugs [SSRIs], exposure to pollution, and maternal infection and/or fevers during the first trimester [8, 9, 10]. While advances in diagnostic criteria and awareness have contributed to the rise in ASD diagnoses, the disorder remains diagnosed solely through behavioral observation [5, 7]. This reflects the persistent lack of molecular biomarkers, extraordinary complexity, and diversity of its underlying biology [11]. Moreover, comorbidities such as epilepsy, gastrointestinal disturbances, and sensory processing disorders often exacerbate the clinical burden and diminish the quality of life [12, 13]. Existing therapies are largely symptomatic, targeting irritability and behavioral issues rather than the root causes of the disorder, underscoring the urgent need for understanding the biology of ASD [7].

Neurodevelopmental models increasingly implicate impaired neuronal maturation and aberrant synaptic pruning as contributing factors in ASD etiology [14]. During early postnatal development, the brain generates an abundance of synaptic connections, which are subsequently refined through activity‐dependent pruning to establish efficient and adaptable neural circuits [15, 16]. Microglia, the brain's resident immune cells, are central to this process [15]. They regulate the elimination of excess synapses, modulate dendritic remodeling, and help balance excitatory and inhibitory signaling within the Central Nervous System (CNS) [17, 18]. Dysregulation of microglial activity, including impaired synaptic engulfment and disrupted neuron‐microglia signaling (e.g., via the CX3CL1–CX3CR1 axis [C‐X3‐C motif chemokine ligand 1 and receptor 1]), has been implicated in several ASD models and is associated with behavioral phenotypes characteristic of the disorder [19]. These findings emphasize the critical importance of microglial‐neuronal communication during early brain development.

At the molecular level, disruptions in the expression of neuronal maturation markers, such as Tuj1 (Class III β Tubulin), MAP2 (Microtubule‐Associated Protein 2), and synapsin‐1, along with abnormal microglial phenotypes marked by altered TMEM119 (Transmembrane Protein 119) and TGFβ1 (Transforming Growth Factor β1) expression, have been observed in neurodevelopmental disorder patient tissue and animal models [20, 21, 22, 23, 24]. Together, these data point to a broader failure of neuro‐glial crosstalk, contributing to the developmental deviations observed in ASD patients.

In parallel with defective pruning, mitochondrial dysfunction has emerged as a key contributor to the pathogenesis of ASD. Beyond their role in cellular energy production, mitochondria are now recognized as central regulators of neuronal proliferation, differentiation, and synapse formation [25, 26]. The high metabolic demands of the developing brain require efficient mitochondrial oxidative phosphorylation (OXPHOS) [25]. Yet, individuals with ASD often exhibit impaired mitochondrial function, as evidenced by reduced ATP levels, dysregulated electron transport chain (ETC) activity, elevated oxidative stress, and abnormal metabolite profiles, such as increased lactate to pyruvate levels [27, 28]. Further, disruptions in mitochondrial dynamics, including an imbalance between fission and fusion [25], lead to fragmented mitochondria, ROS accumulation, and neuronal dysfunction [29], hallmarks also observed in postmortem ASD brain tissue [30].

Microglia‐neuron metabolic crosstalk has recently emerged as a novel mechanistic axis implicated in neurodevelopmental disorders. Microglia are capable of transferring functional mitochondria to stressed neurons, modulating metabolic profiles, and enhancing mitochondrial plasticity. These findings suggest that microglia contribute to neuronal development through synaptic remodeling and directly influence neurons' bioenergetic capacity. The precise mechanism of this support, whether through physical contact, secreted vesicles, or metabolic intermediates, remains poorly understood, particularly in the context of neurodevelopment.

Despite growing mechanistic insights, it remains unclear whether physical cell‐cell contact between microglia and neurons is required for these beneficial effects or whether microglial‐derived mitochondria and metabolites can independently influence neuronal maturation. To address this knowledge gap, we utilized a human 3D in vitro model of the brain [31] to investigate the role of microglial mitochondrial support in neuronal development. Specifically, we evaluated 1) whether direct microglia‐neuron interaction is necessary for promoting neuronal maturation, 2) the functional impact of mitochondrial transfer on neuronal bioenergetics, and 3) how our model recapitulates in vivo‐like neurodevelopmental features in ASD patients. We found that treatment with microglial mitochondria partially promotes neuronal maturation and induces transcriptional alterations as early as 48 h post‐treatment. However, full developmental support requires additional microglia‐derived signals and direct neuron–microglia interactions. Together, these findings provide mechanistic insight into how microglia contribute to neuronal maturation and point to mitochondrial transfer as a potential pathway for understanding the cellular basis of ASD.

## 2 Results

### 2.1 Microglia Promote Neuronal Maturation and Network Development in a 3D Human In Vitro Model

**FIGURE 1:**
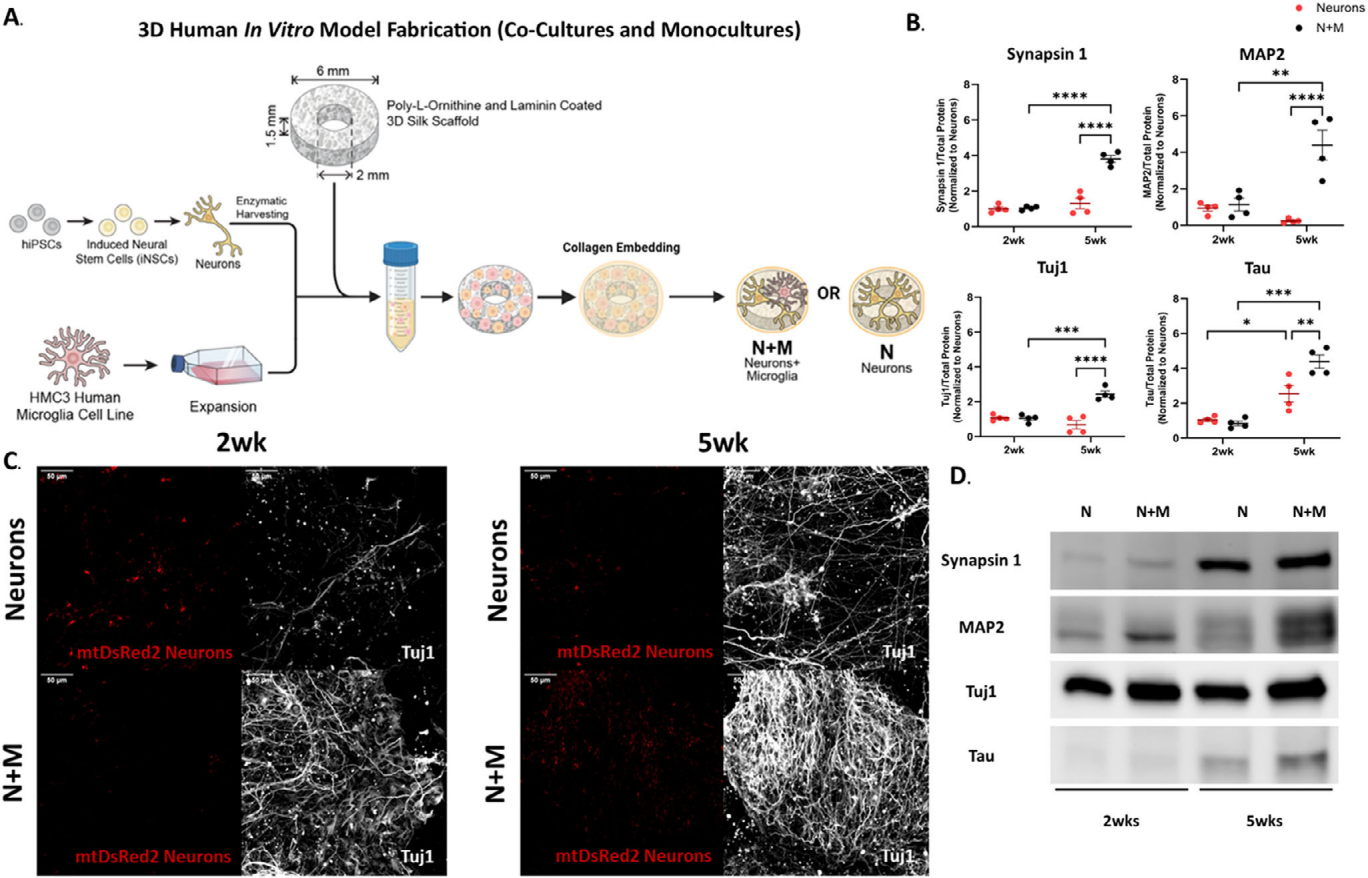
Microglia boosts Neuronal Maturation—(A) Schematic representation of the preparation of our NM co‐culture and neuron monoculture. (B) Western blot quantification of select neuron maturation markers (Synapsin 1, MAP2, Tuj1, and TAU [Tubulin Associated Unit]) with Neurons in red and NM in Black. *n* = 4 for NM and Neurons. Two‐way ANOVA looking at effects of time and culture type with Tukey post‐hoc test and α = 0.05. Grubbs’ outlier analysis method: no outliers were removed. Analysis completed in GraphPad Prism. (C) Representative images of Tuj1 and mtDsRed2 neuron mitochondria. Nikon A1 inverted LUNV confocal microscope 20x objective 1024 × 1024 pixels with 50‐steps Max projection. Scale bar: 50 µm.

To explore the benefits of microglia in promoting neuronal maturation, we used our 3D in vitro human tissue model composed of induced neuronal stem cells (iNSCs) and an HMC3 microglia cell line with fluorescently tagged mitochondria (dsRED2 and EBFP2, respectively) enabling direct comparison of Neuron only monocultures (N) and Neuron + Microglia (NM) co‐cultures to naïve neuron monocultures (Figure 1A) [31, 32]. We analyzed the presence of neuron‐specific maturation markers at 2 and 5‐week post‐seeding to demonstrate the developmental differences between the two experimental conditions.

At 2 weeks post‐seeding, we observed no statistical difference in the expression of Synapsin 1, MAP2, Tau (Tubulin Associated Unit), or Tuj1 between neurons and NM conditions (*p*‐value > 0.05). In contrast, by 5‐week, NM co‐cultures exhibited significant upregulation of the above‐mentioned maturation markers compared to neurons (*p*‐value < 0.0001 for Synapsin 1, MAP2, and Tuj1, *p*‐value < 0.01 for Tau). These results suggest accelerated and more robust neuronal maturation in the presence of microglia.

Immunofluorescence analysis further confirmed enhanced Tuj1 expression and extensive neuronal network formation in NM cultures at both time points, with pronounced network complexity evident by 5 weeks (Figure 1C). While network structures were present in neuron‐only cultures, NM co‐cultures showed greater neurite density and connectivity, suggesting more advanced functional integration. At last, the interconnectivity of the mitochondrial network also visually increased at 5‐week in the NM cultures compared to the naïve neuron condition. Mitochondrial networks are crucial in the functionality of cells like neurons that rely on high levels of ATP. Together, these results support a critical role for microglia in orchestrating neuronal maturation and network assembly, potentially through both trophic and metabolic mechanisms.

### 2.2 Microglia Mitochondria Reprogram Neuronal Bioenergetics in a 3D Human Brain Model

**FIGURE 2:**
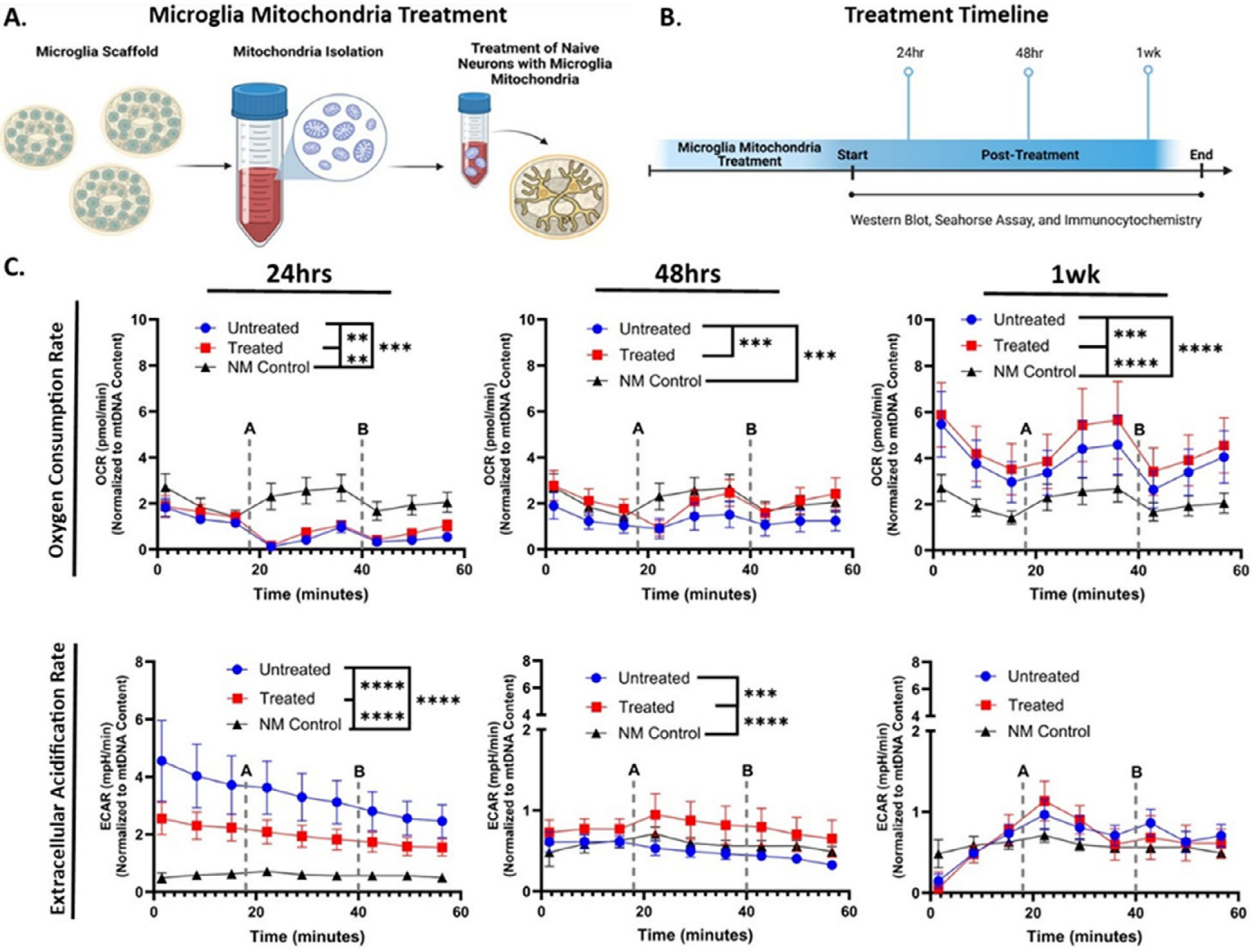
Mitochondria Enhance Neuronal Bioenergetics over time. (A) Schematic representation of the experimental design. (B) Treatment and analysis timeline. (C) Real‐Time ATP Rate Assay results comparing OCR and ECAR between different conditions across select time points. *n* = 9 scaffolds NM and Untreated. *n* = 12 for Treated. Mean ± SEM. One‐way repeated measures ANOVA with Dunnett's post‐hoc test, *p*‐value < 0.05, α = 0.05. ROUT outlier analysis method, no outliers were removed. Analysis completed in GraphPad Prism. Each timepoint was replicated at least twice.

To begin dissecting the mechanism underlying this microglia‐driven maturation, we next considered whether the transfer of microglial mitochondria could contribute to the observed neuronal development. Live‐cell imaging revealed dynamic movement of microglial mitochondria into neurons (Video S1), supporting the possibility that organelle transfer provides an additional layer of trophic support. Thus, we hypothesized that mitochondrial transfer itself may be a critical driver of neuronal maturation. To determine the contribution of microglial mitochondria in neuronal bioenergetic maturation, we isolated fresh microglial mitochondria from 3D microglia monocultures and applied them directly to naïve neuronal cultures. Bioenergetic profiling was performed at 24 and 48 h, and 1‐week post‐treatment using Seahorse XF analysis to measure Oxygen Consumption Rate [OCR] and Extracellular Acidification Rate [ECAR]) (Figure 2A,B).

Mitochondria transfer induced significant alterations in neuronal metabolic activity. At 24 h, OCR was significantly elevated in treated neurons compared to NM (*p* < 0.01), and untreated neurons (*p* < 0.001), indicating a rapid enhancement in oxidative phosphorylation. This trend persisted at 48 h, where OCR in the treated group remained comparable to NM (*p*‐value > 0.05), suggesting metabolic stabilization. By 1‐week, the Treated condition exhibited the highest OXPHOS activity across conditions (*p*‐value < 0.0001 vs NM, *p* < 0.001 vs untreated), while untreated neurons also showed increased OCR relative to NM (*p* < 0.0001), likely reflecting compensatory stress responses.

ECAR analysis further supported a metabolic shift. At 24 h, untreated neurons displayed elevated ECAR compared to both treated and NM (*p* < 0.0001), indicative of heightened glycolysis. However, by 48 h, ECAR significantly increased in treated neurons (*p* < 0.001 vs. Untreated; *p* < 0.0001 vs. NM), aligning with enhanced glycolytic activity. These effects resolved by 1 week, with no significant differences in ECAR among groups.

Collectively, these findings demonstrate that treatment with microglial mitochondria can modulate neuronal bioenergetics, enhancing both oxidative and glycolytic capacity in a temporally dynamic manner. This suggests that microglial mitochondrial transfer may be a potential regulatory mechanism in neuronal metabolic maturation.

### 2.3 Microglial Mitochondria Restore Neuronal Mitochondrial Dynamics

**FIGURE 3:**
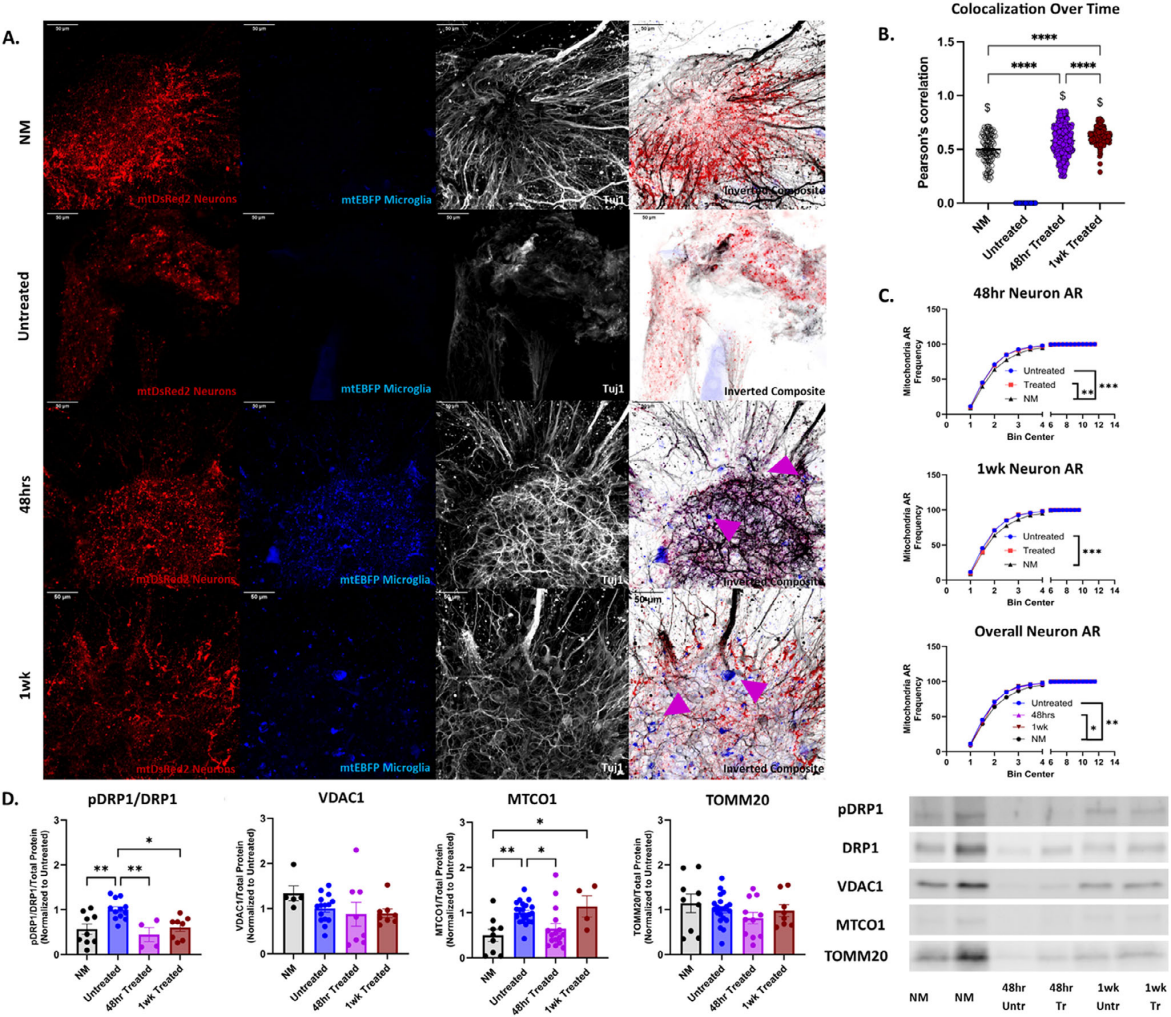
Mitochondrial Health and Colocalization: (A) Representative images of NM, Untreated, 48 h Treated, and 1‐week Treated. mtDsRed2 neurons and mtEBFP2 microglia. Tuj1 staining for neuronal networks. Nikon A1 inverted LUNV confocal microscope, 20x objective, 1024 × 1024 pixels, with 50‐steps Max projection. Scale bar: 50 µm. (B) Colocalization analysis was performed with JaCoP using Pearson's Correlation Coefficient. For each image stack, colocalization between mtDsRed2‐positive mitochondria and mtEBFP‐positive microglial mitochondria was quantified across all 50 individual z‐slices. $ denotes significantly different than Untreated. At least *n* = 9 scaffolds NM and Untreated. *n* = 12 for Treated. Mean ± SEM. One‐way ANOVA with Tukey post‐hoc test, *p*‐value < 0.05, α = 0.05. ROUT outlier analysis method, outliers removed. Each timepoint was replicated at least twice. (C) Mitochondria aspect ratio comparing the length and width of mitochondria at select time points (48 h and 1‐week) along with NM and Untreated. At least *n* = 6 scaffolds NM, *n* = 9 for Untreated, and *n* = 6 for Treated. Mean ± SEM. Kruskal‐Wallis with Dunn's post‐hoc test, *p*‐value < 0.05, α = 0.05. ROUT outlier analysis method, no outliers were removed. Each timepoint was replicated at least twice. (D) Mitochondria‐specific health markers, pDRP1/DRP1, TOMM20, VDAC1, and MTCO1 intracellular mitochondria western blots normalized to total protein concentration per lane and to the Untreated condition. *n* = 9 for NM and Untreated. *n* = 12 for 48 h and 1‐week Treated. Mean ± SEM. One‐way ANOVA with Tukey post‐hoc test, *p*‐value < 0.05, and α = 0.05. Each timepoint was replicated at least twice. ROUT outlier analysis method, outliers were removed for pDRP1/DRP1 (48 h Treated and 1‐week Treated), VDAC1 (NM, 48 h Treated, 1‐week Treated), MTCO1 (1‐week Treated), and TOMM20 (1‐week Treated). Analysis completed in GraphPad Prism.

In the NM co‐cultures, microglial and neuronal mitochondria show clear colocalization (Figure 3A). While there were alterations on a bioenergetic level at 24 h, there were no significant alterations to protein expression or mitochondrial health at said timepoint. Due to this, the 24 h timepoint was removed from analysis (Figure S2). By 48 h post‐treatment, we observed visual confirmation of microglia mitochondria integrating into neurons, a trend that persisted through the 1‐week time point, supporting the ability of neurons to internalize extracellular mitochondria for potential metabolic support [33]. Pearson's correlation coefficients for colocalization of microglial mitochondria were significantly different at both the 48 h and 1‐week timepoint, and all conditions had increased colocalization in comparison to Untreated (Figure 3B).

Quantitative analysis of mitochondrial morphology revealed that, at 48 h, neurons treated with microglial mitochondria exhibited significantly fragmented mitochondria (reduced aspect ratio [AR]) compared to NM (*p* < 0.01), consistent with a fragmented mitochondrial state. However, by 1‐week post‐treatment, AR values in treated neurons no longer differ from NM, indicating a restoration of mitochondrial morphology. In contrast, untreated neurons retained a significantly lower AR compared to both 48 h and 1‐week treated groups (*p* < 0.001), suggesting persistent mitochondrial fragmentation in the absence of treatment (Figure 3C).

Lastly, to assess mitochondrial health, we examined markers of fission and function. Phosphorylation of DRP1 (Dynamin‐Related Protein 1) at serine 616 (a key marker of mitochondrial fission) was significantly elevated in untreated neurons compared to NM (*p* < 0.01), 48 h treated (*p* < 0.01), and 1‐week treated groups (*p* < 0.05), supporting the AR findings (Figure 3D). While TOMM20 (Translocase of the Outer Mitochondria Membrane 20) and VDAC1 (Voltage‐Dependent Anion Channel 1) levels remained unchanged across conditions, intracellular MTCO1 (CCMitochondrially Encoded Cytochrome C Oxidase I) expression was highest in untreated neurons (vs. NM, *p* < 0.01; vs. 48 h treated, *p* < 0.05). Interestingly, 1‐week treated neurons showed increased MTCO1 compared to NM (*p* < 0.05), suggesting potential compensatory mitochondrial activity at later timepoints. Together, these data suggest that treatment with microglial mitochondria promotes uptake into neurons and supports improved mitochondrial dynamics, specifically by reducing fission and restoring healthier mitochondrial morphology.

### 2.4 Microglial Presence Enhances Neurodevelopment beyond Mitochondrial Transfer

**FIGURE 4:**
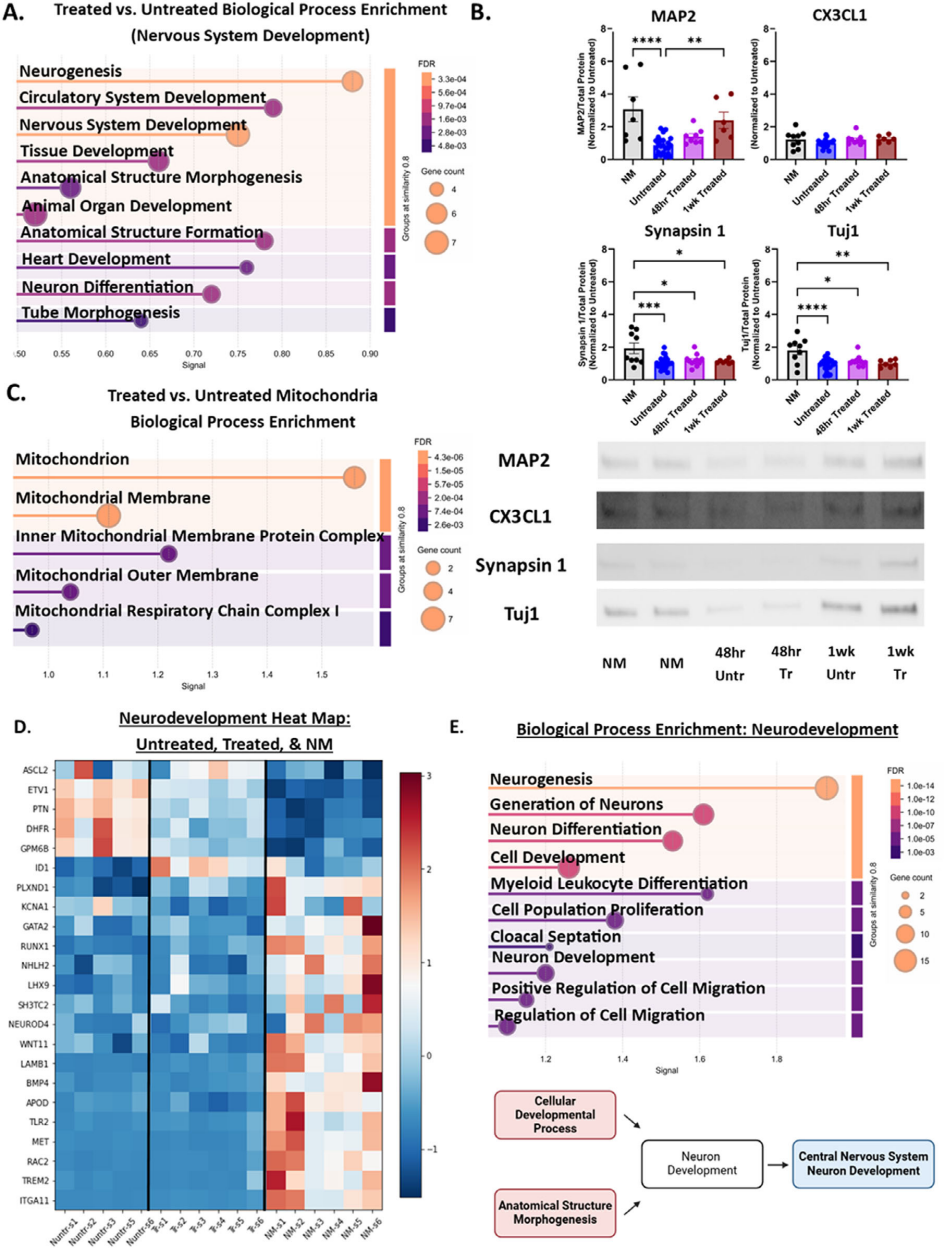
Treatment Effect on Nervous System Development and Mitochondria: (A) Biological process enrichment, specifically targeting nervous system development between treated and untreated. Due to wanting to see the finer details in the differences between the treated and untreated conditions, the Log_2_(Fold Change) threshold was set to 0.2 for analysis. However, an adjusted p‐value of < 0.05 was still required to be deemed “significant”. Medium confidence interaction score (0.4). False Discovery Rate (FDR) ≤ 0.05. (B) Neuronal maturation markers MAP2, CX3CL1, Synapsin 1, and Tuj1 over time, western blots normalized to total protein concentration per lane and to the Untreated condition. (C) Biological process enrichment, specifically mitochondria‐related genes from Mito Carta 3.0, between treated and untreated. Log_2_(Fold Change) = 0.2, adjusted *p*‐value of < 0.05, medium confidence interaction score (0.4), and FDR ≤ 0.05 [34]. (D) Comparison Between NM vs. Treated Heat map of z‐score normalized DEGs comparing NM with 48 h treated scaffolds. *p*‐value < 0.05 required to be deemed significant for further analysis. Medium confidence interaction score (0.4). False Discovery Rate (FDR) ≤ 0.05. (E) Biological process enrichment focusing on neurogenesis between NM and 48 h Treated. Log_2_(Fold Change) = 1.0, adjusted *p*‐value of < 0.05, medium confidence interaction score (0.4), and FDR ≤ 0.05. Mean ± SEM. One‐way ANOVA with Tukey post‐hoc test, *p*‐value < 0.05, and α = 0.05. ROUT outlier analysis method. Analysis completed in GraphPad Prism. *n* = 3 for NM and Untreated. *n* = 4 for 48 h and 1week Treated. Each timepoint was replicated at least twice.

To investigate whether the early bioenergetic changes induced by microglial mitochondria are accompanied by transcriptional reprogramming, we performed RNA sequencing on neurons treated for 48 h with microglial mitochondria and compared them to untreated controls. Differential gene expression analysis revealed significant enrichment of nervous system development pathways, including neurogenesis (FDR = 0.0041), nervous system development (FDR = 0.0033), and neuron differentiation (FDR = 0.0198), all with adjusted p‐values < 0.05 and log_2_(fold change) ≥ 0.2 (Figure 4A). Among the upregulated genes were ID1 (Inhibitor of DNA Binding 1), ID3 (Inhibitor of DNA Binding 3), and SALL4 (Spalt Like Transcription Factor 4), which regulate proliferation and differentiation, as well as RUNX1 (Runt Related Transcription Factor 1), LAMB1 (Laminin Subunit Beta 1), PLXND1 (Plexin D1), and MED12 (Mediator Complex Subunit 12), which are associated with neuronal development. These transcriptional changes at 48 h preceded phenotypic differences observed at 1 week, when treated neurons showed increased MAP2 expression, a marker of dendritic maturation, compared to untreated controls (*p* < 0.0001). To confirm that treatment did not induce neurodegenerative changes, we compared Tuj1‐positive neural networks and examined the expression of key neurodegenerative markers, including amyloid precursor protein (APP), transactive response DNA‐binding protein 43 (TDP‐43), and phosphorylated tau (pTau), alongside NM co‐cultures. No adverse effects on neuronal networks or neurodegenerative markers were detected (Figure S3). In addition, genes linked to negative regulation of nervous system development were enriched, driven by the upregulation of PTN (Pleiotrophin) and ASCL2 (Achaete‐Scute Family BHLH Transcription Factor 2) in treated neurons, and TLR2 (Toll‐Like Receptor 2) and TREM2 (Triggering Receptor Expressed on Myeloid Cells 2) in NM. The latter two are microglia‐associated and likely reflect broader functions of intact microglia beyond mitochondrial transfer, including their influence on neuroimmune signaling and developmental pruning (Figure 4D).

Focusing on mitochondrial‐specific genes from the MitoCarta 3.0 database, we identified seven genes significantly altered by treatment (Figure S4). These genes were enriched in pathways related to the inner mitochondrial membrane protein complex (FDR = 0.0062) and mitochondrial respiratory chain complex I (FDR = 0.0265) and were predominantly downregulated in treated neurons. The only upregulated mitochondrial gene was CPT1B, a key regulator of fatty acid oxidation. Despite downregulation of ETC‐related genes, neurons treated with microglial mitochondria demonstrated increased oxidative metabolism (Figure 2C), suggesting a compensatory shift toward fatty acid oxidation. These results suggest that while neurons incorporate extracellular mitochondria, this may be accompanied by transcriptional remodeling that attenuates endogenous ETC gene expression.

To determine whether mitochondrial transfer alone could replicate the effects of direct microglia‐neuron interaction, we compared the transcriptional profiles of the 48 h Treated condition to the NM co‐culture. Although mitochondrial treatment promoted neurodevelopmental gene expression relative to the Untreated condition, NM cultures exhibited significantly higher expression of pathways related to neuron generation (FDR = 7.92e‐10), neuron differentiation (FDR = 16.92e‐9), and neuron development (FDR = 9.09e‐6), indicating a more robust neurogenic program (Figure 4E). The NM co‐culture is also superior to the Untreated and Treated conditions from a mitochondrial function perspective (Figure S3). Untreated neurons, while focusing on OXPHOS‐based metabolism, showed enrichment of abnormal mitochondrial function pathways and ETC defects. Treatment with microglial mitochondria shifted metabolism away from OXPHOS to glycolysis, bolstering our ECAR data from Figure 2. The NM co‐culture was more focused on protein production, with the condition being positively associated with peptide chain elongation and translation. Overall, mitochondrial function was positively affected by treatment, with enrichment of glycolysis‐ and protein‐production–related pathways. However, the NM co‐culture allows for a balanced metabolic environment and supports efficient mitochondrial function.

To further dissect microglial contributions beyond direct mitochondrial transfer, we next tested whether soluble cues alone could influence neuronal maturation. Treatment of neurons with microglia‐conditioned media lacking mitochondria produced a rapid and pronounced increase in MAP2 within 48 h, indicating that microglial secreted factors can independently promote early dendritic elaboration without the need or participation of mitochondrial transfer (Figure S5A). CX3CL1 expression also increased after one week, consistent with delayed engagement of neuron‐microglia communication pathways. In contrast, Synapsin‐1 levels were reduced relative to NM co‐cultures, and mitochondrial markers exhibited a dysregulated profile, including elevated pDRP1, reduced TOMM20, and a biphasic VDAC1 response, suggesting that secreted cues do not support the mitochondrial‐dynamic remodeling associated with maturation (Figure S5B). Multiple‐dose inhibition of mitochondrial transfer in NM cultures using Rasarfin or Ciliobrevin yielded a highly similar phenotype (Figure S6A,B). Despite effective suppression of organelle exchange, MAP2 levels increased beyond those of NM cultures, whereas Synapsin‐1 and CX3CL1 remained reduced (Figure S6A). Mitochondrial markers again shifted toward an immature or fragmented state, with elevated pDRP1 and reduced MTCO1 (Figure S6B).

We next asked how mitochondrial treatment relates to ASD‐associated biology when pathway enrichment is restricted to a manually curated set of ASD‐related terms. In Figure S7, lollipop plots show pathways altered between mitochondria‐treated neurons (NTr) and untreated neurons (NUntr), displayed as “convergent” (left; same direction reported in ASD literature) and “divergent” (right; opposite direction). Along the convergent axis, NTr engages innate immune/inflammatory signaling, as well as selected extracellular matrix (ECM) remodeling modules (as labeled in the plot). Along the divergent axis, NTr shows opposite regulation for structural and metabolic programs, covering ECM biogenesis/modification and glycosaminoglycan/proteoglycan pathways, ion/nutrient transport, and sensory/GPCR‐linked processes (as labeled), indicating overlap with ASD primarily on immune axes and opposition along structural and metabolic axes.

Collectively, these findings indicate that mitochondrial transfer alone can induce transcriptional shifts in neurons that overlap with ASD biology along immune and ECM axes, while simultaneously diverging from ASD on structural, metabolic, and synaptic programs. This selective pattern suggests that microglial mitochondria can partially reproduce ASD‐relevant immune signaling but, at the same time, promote pathways that counteract the downregulation observed in ASD, particularly those linked to neuronal maturation and mitochondrial function. Together, this analysis underscores that mitochondrial transfer shapes neuronal identity in a manner distinct from the broader influence of intact microglia, highlighting both its potential mechanistic contribution and its limits as a standalone driver of neuronal development.

### 2.5 Microglia Drive ASD‐Like Immune Convergence, While Both Microglia and Mitochondria Diverge on Maturation and Epigenetic Programs

**FIGURE 5:**
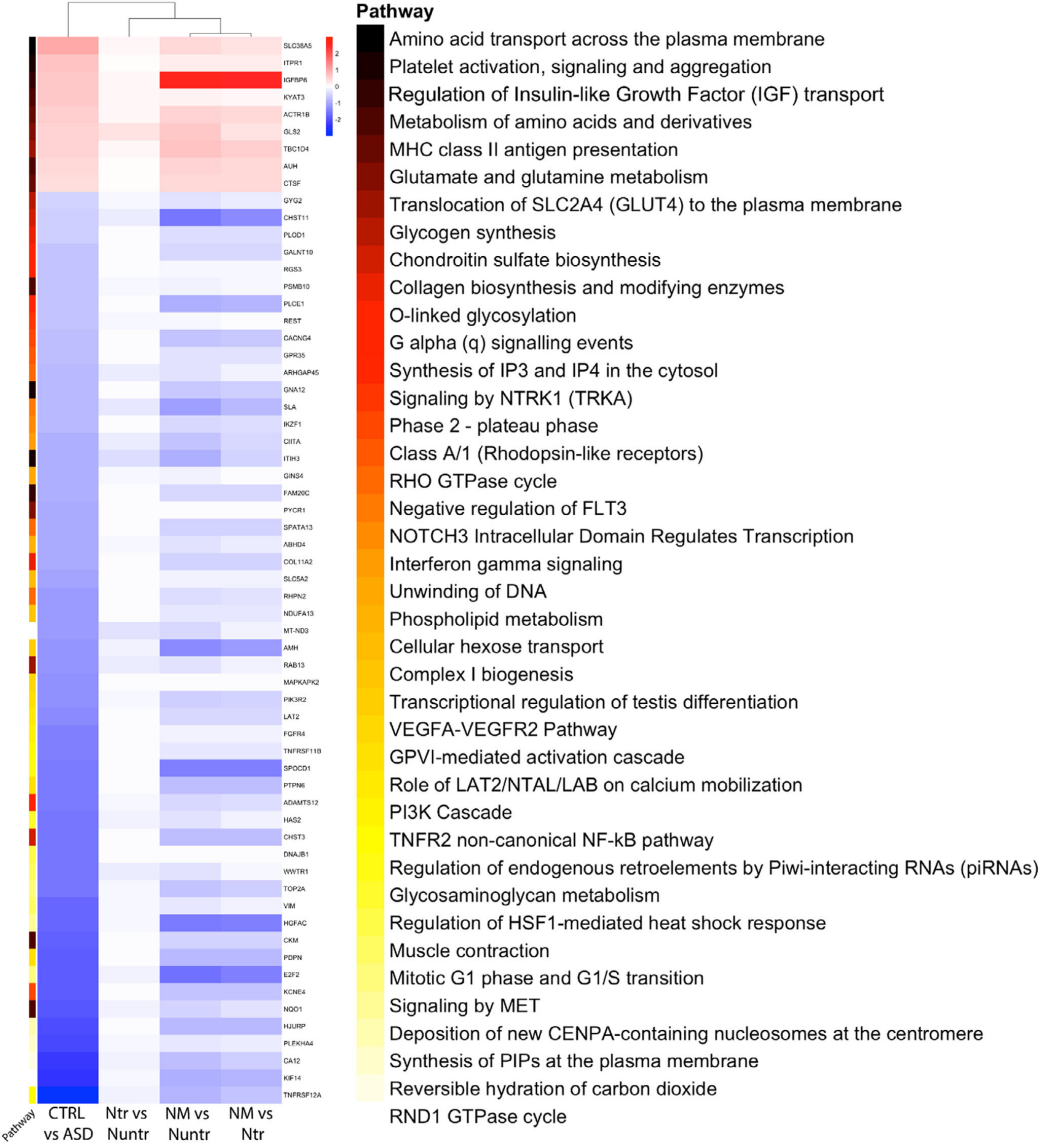
Pathway enrichment analysis reveals microglia as the primary driver of transcriptomic convergence with ASD patient signatures. Heatmap of enriched pathways comparing ASD patient cortex to neuronal cultures treated for 48 h with microglial mitochondria (NTr), neuron–microglia co‐cultures (NM), and untreated neuron monocultures (CTRL). Pathway enrichment analysis between ASD patients, mitochondria‐treated, and NM groups. Log_2_(Fold Change) = 0.2, adjusted *p*‐value of < 0.05, medium confidence interaction score (0.4), and FDR ≤ 0.05 [34].

At last, we directly compared the 48 h RNA‐seq profiles of mitochondria‐treated neurons (NTr) and neuron–microglia co‐cultures (NM) with the ASD patient cortex (Figure 5; Figures S7 and S8) [35]. Pathway enrichment analysis revealed that NM aligned with ASD across immune and extracellular‐matrix programs, including Toll‐like receptor signaling (TLR2, Toll‐like receptor 2; TLR4, Toll‐like receptor 4), TNF/NF‐κB signaling (TNF, tumor necrosis factor; RELA, NF‐κB p65 subunit; NFKBIA, NF‐κB inhibitor alpha), interferon and cytokine responses (IFNG, interferon gamma; IL1B, interleukin‐1 beta; CXCL10, C‐X‐C motif chemokine ligand 10), Fc‐receptor and complement‐linked terms (FCGR2A, Fc gamma receptor IIa; C1QA, complement component 1q subcomponent subunit A; C3, complement component 3), integrin–RHO cytoskeletal remodeling (ITGA4, integrin alpha 4; RHOA, Ras homolog family member A; ROCK2, Rho associated coiled‐coil containing protein kinase 2), and extracellular matrix biosynthesis such as collagen and glycosaminoglycan pathways (COL1A1, collagen type I alpha 1 chain; COL4A1, collagen type IV alpha 1 chain; ACAN, aggrecan). Growth‐factor signaling cascades, including VEGF/PI3K–AKT (VEGFA, vascular endothelial growth factor A; PIK3CA, phosphatidylinositol‐4,5‐bisphosphate 3‐kinase catalytic subunit alpha; AKT1, AKT serine/threonine kinase 1) and nutrient/transport processes (SLC2A1, solute carrier family 2 member 1/GLUT1; SLC7A5, solute carrier family 7 member 5) were also aligned with ASD. These convergent pathways were predominantly downregulated in ASD, suggesting that microglial presence reproduces the immunosuppressive and ECM‐remodeling patterns characteristic of the disorder (Figure 5; Figure S8). In contrast, NTr showed minimal convergence on these modules and remained closer to untreated neurons, indicating that mitochondria alone are not sufficient to drive ASD‐like immune alignment and may instead confer a degree of resistance against maladaptive remodeling.

Additional analyses (Figure S8) identified pathways that diverged from ASD, with both NTr and NM shifting away from the patient state on neuronal maturation and synaptic programs, including neurogenesis (NEUROD1, neuronal differentiation 1; DCX, doublecortin), synapse assembly and transmission (DLG4, discs large MAGUK scaffold protein 4/PSD95; SYN1, synapsin I), vesicle cycling (VAMP2, vesicle associated membrane protein 2; SNAP25, synaptosome associated protein 25), and axon–dendrite organization (MAP2, microtubule associated protein 2; TUBB3, tubulin beta 3 class III). Divergence was also evident in chromatin regulation (HDAC2, histone deacetylase 2; CREBBP, CREB binding protein), cell‐cycle modules (CCND1, cyclin D1; CDK4, cyclin dependent kinase 4), calcium handling (CAMK2A, calcium/calmodulin dependent protein kinase II alpha; GRIN2B, glutamate ionotropic receptor NMDA type subunit 2B), and mitochondrial biogenesis and translation (TFAM, transcription factor A mitochondrial; POLG, DNA polymerase gamma; MRPL12, mitochondrial ribosomal protein L12). In the ASD cortex, these modules are broadly downregulated, whereas both NTr and NM upregulate these gene sets relative to ASD, indicating movement away from the ASD state and partial restoration of maturation‐ and mitochondria‐linked programs.

Together, these results suggest that microglia are the primary driver of ASD‐like convergence on immune and extracellular‐matrix pathways (consistent with their suppression in ASD), while both experimental conditions diverge from ASD on neuronal development, chromatin/cell‐cycle, calcium, and mitochondrial biogenesis/translation by increasing these programs relative to ASD. The minimal immune convergence in mitochondria‐treated neurons suggests that exogenous mitochondria may provide partial protection against ASD‐like transcriptomic alignment.

## 3 Discussion

### 3.1 Metabolic Reprogramming Reflects Developmental Maturation

Our data suggest that the exogenous delivery of microglial mitochondria to neurons induces a temporal shift in metabolic activity that closely resembles developmental transitions observed in human neural maturation [36]. At 48 h post‐treatment, we observed a normalization of both oxidative phosphorylation (OXPHOS) and extracellular acidification rate (ECAR) to levels comparable to neuron‐microglia (NM) co‐cultures. By one week, OXPHOS activity was substantially elevated while ECAR remained unchanged, indicating a durable metabolic reprogramming toward aerobic respiration. This resembles patterns observed in human stem cell‐derived neurons, where a developmental switch from glycolysis to oxidative metabolism is essential for neuronal differentiation, dendritic growth, and synaptic activity [18, 37, 38]. These results are consistent with the possibility that mitochondrial function contributes to establishing neuronal metabolic identity.

Recent studies have highlighted that mitochondrial state and metabolic remodeling are closely linked to the timing and progression of human development [39, 40], supporting the concept that mitochondria participate in regulating developmental tempo. In parallel, metabolic coupling between microglia and neurons has been shown to support neuronal function [41], and tunneling nanotube connections can enable transfer of healthy microglial mitochondria to neurons under stress [42]. Building on this framework, our results suggest that microglial mitochondria contribute to a form of maturational gating in which the developmental trajectory of neuronal mitochondria, and in turn neuronal maturation, depends in part on microglial mitochondria input.

Finally, when considering a context of compromised neuronal development such as ASD, our comparison indicates that mitochondrial‐driven maturation programs shift away from ASD‐associated deficits, while microglia‐dependent immune and ECM pathways show partial alignment with ASD‐related signatures (Figure 5; Figures S7 and S8). Thus, our results suggest that while key mechanisms of healthy neuronal maturation are becoming clearer, microglial mitochondrial regulation may represent one potential pathway to consider in understanding altered developmental trajectories in ASD.

### 3.2 Transcriptomic Shifts Support Neurodevelopmental Progression

RNA sequencing suggested that mitochondrial treatment alone engages a subset of transcriptional programs associated with nervous system development, including neurogenesis, neuron differentiation, projection development, and both central and peripheral nervous system formation. Genes involved in mitochondrial structure and function, particularly membrane‐bound respiratory complexes, were also significantly upregulated. These findings are consistent with developmental studies implicating mitochondrial biogenesis and cristae remodeling as key drivers of neural lineage specification [43]. Compared to NM conditions, treated neurons continued to show enrichment in developmental pathways, including axis elongation and neurogenesis, suggesting that mitochondrial transfer provides a unique and sustained maturation signal.

When placed in the context of ASD, however, the implications become more nuanced. At 48 h, NM aligned with ASD across immune and extracellular‐matrix axes (for example, Toll‐like receptor and TNF/NF‐κB signaling, interferon/cytokine responses, complement/Fc‐receptor terms, integrin‐RHO remodeling, and collagen/ECM organization), whereas mitochondria‐treated neurons showed minimal convergence on these pathways. This suggests that while intact microglia accelerate neuronal maturation, they may also engage immune remodeling programs that overlap with those reported in ASD transcriptomic datasets. In contrast, both NTr and NM diverged from ASD on neuronal maturation and synaptic programs (neurogenesis, synapse assembly/transmission, vesicle cycling, axon/dendrite organization), as well as chromatin/cell‐cycle modules, calcium handling, and mitochondrial biogenesis/translation. In the ASD cortex, these pathways are broadly downregulated; the fact that both experimental conditions increased them relative to ASD indicates a movement away from the ASD state and partial restoration of maturation‐ and mitochondria‐linked functions (Figure 5; Figures S7 and S8).

Mitochondrial transfer can influence neuronal maturation pathways that are altered in ASD, while functioning as one element within a broader and multifactorial microglial response. Our data support a distinction between two separate dimensions of microglia‐neuron interaction: mitochondrial‐mediated regulation of neuronal metabolic and transcription maturation, and microglia‐driven immune and extracellular‐matrix signaling. These processes appear to contribute differently to developmental outcomes, suggesting that modulation of mitochondrial input may affect neuronal maturation independently of other microglial functions.

The demonstration that isolated microglial mitochondria are sufficient to alter neuronal metabolic and transcriptional programs places our results within a growing body of work exploring mitochondria as functional regulators of neuronal health [44, 45, 46, 47]. In this context, mitochondrial transplantation has been investigated in several neuronal conditions associated with mitochondrial dysfunction [47]. For example, in a preclinical model of pilocarpine‐induced status epilepticus in mice, delivery of autologous mitochondria was associated with reduced neuronal loss, altered metabolite profiles, and decreased reactive oxygen species [48]. Although such studies have not been conducted in ASD, they illustrate that manipulation of mitochondrial state can meaningfully influence neuronal outcomes. Integration of our in vitro functional data with patient transcriptomic analyses, therefore, provides a conceptual framework for considering how microglial mitochondrial regulation may intersect with developmental processes relevant to ASD.

### 3.3 Structural and Functional Implications of Mitochondrial Transfer

While metabolic and transcriptional indicators of maturation were strongly enhanced, not all structural features of maturation reached the level observed in neuron‐microglia (NM) co‐cultures. At the protein level, mitochondrial treatment increased expression of MAP2, consistent with enhanced dendritic elaboration [49, 50]. In contrast, Synapsin‐1 and Tuj1 levels remained largely unchanged, indicating that synaptogenesis and cytoskeletal integration likely require additional microglia‐derived signals beyond mitochondrial input alone. These observations suggest that mitochondrial transfer supports specific dimensions of neuronal maturation but is not sufficient to drive the full structural and synaptic maturation program. The ASD comparison supports this interpretation: mitochondria‐treated neurons exhibited transcriptomic shifts away from ASD‐associated deficits in maturation‐linked programs, whereas microglia‐driven immune/ECM pathways showed partial alignment with ASD‐related signatures at 48 h. This separation highlights that distinct components of microglial support (mitochondrial vs. immune/secreted signaling) can influence different axes of neuronal maturation.

### 3.4 Differential Microglial Inputs Modulate Distinct Dimensions of Neuronal Maturation

In addition to the mitochondrial‐specific effects, our perturbation experiments revealed that microglial secreted factors and mitochondrial transfer contribute distinct and potentially complementary aspects of neuronal maturation. Secretome‐only treatment was sufficient to induce a rapid increase in MAP2, indicating that certain structural components of maturation can proceed in the absence of mitochondrial transfer. However, this structural elaboration did not coincide with increases in Synapsin‐1 or CX3CL1, and mitochondrial‐dynamics markers remained fragmented or dysregulated, suggesting that soluble cues alone do not support the coordinated metabolic and synaptic maturation observed in NM co‐culture. Consistent with this interpretation, pharmacological inhibition of mitochondrial transfer in NM cultures reproduced a similar dissociation: MAP2 increased beyond NM levels, whereas markers of synaptic and mitochondrial maturation remained reduced.

These patterns parallel developmental studies showing that neuronal maturation unfolds in sequential stages, with early dendritic growth preceding later metabolic and synaptic refinement [39, 40, 51]. Within this framework, our data support a model in which microglial secretome and mitochondrial transfer operate through separate but complementary pathways that together enable the full spectrum of microglia‐supported neuronal maturation. This suggests that neuronal maturation may be, at least in part, gated by the maturation state and functional contributions of microglia, with mitochondrial input representing a key regulatory component of this process. Although these findings do not establish causality, they emphasize that coordinated neuronal development requires integrated contributions from both secreted signals and mitochondrial transfer.

### 3.5 Mitochondria as Drivers of Neuronal Maturation

The results of this study support a unifying interpretation in which microglia regulate neuronal maturation through multiple parallel mechanisms. Collectively, these findings add to a growing body of evidence that mitochondria appear to play a central and active role in neuronal maturation [29]. Beyond energy supply, our data suggest that microglial mitochondria can function as biologically active developmental cues that complement other microglial signals, promoting metabolic reprogramming, initiating neurodevelopmental gene expression, and enhancing structural features of neuronal identity. Our results agree with previous studies, which demonstrated the connection to increased mitochondrial activity and accelerated neuronal maturation in human PSC‐derived cortical neurons in vitro. While the full complexity of neuron‐microglia interactions is not replicated by mitochondrial transfer alone, this study provides functional support for the idea that mitochondria may contribute to initiating aspects of the neuronal maturation program, although additional signals are likely required for full maturation. These observations emphasize that mitochondrial transfer represents one component of a broader microglial support system for neuronal development. In the context of the ASD comparison, our data suggest that this mechanism may influence pathways relevant to ASD‐associated biology, but further work will be necessary to determine its translational significance.

## 4 Conclusions and Future Directions

This study identifies microglia and their mitochondria as important regulators of human neuronal maturation. Our findings indicate that microglial mitochondrial transfer selectively promotes metabolic and transcriptional dimensions of maturation, whereas additional microglia‐derived signals are required for full structural and synaptic development. These results support a model in which neuronal maturation is shaped by coordinated inputs from microglia, with mitochondrial contributions acting as one regulatory gate within a broader developmental program.

Comparison with ASD cortical transcriptomic data reveals two separable axes of microglial influence. Mitochondrial‐driven programs shift away from ASD‐associated deficits in neuronal maturation pathways, while microglia‐dependent immune and extracellular‐matrix signals show partial alignment with ASD‐related signatures. This separation suggests that distinct components of microglial support engage different aspects of neurodevelopment, providing a framework for understanding how perturbations of microglial biology may influence specific dimensions of neuronal maturation.

More broadly, microglia emerge as metabolic developmental partners of neurons, indicating that neuronal maturation is not entirely neuron‐autonomous, but depends, in part, on the maturation state and functional contributions of surrounding microglia. This perspective offers a conceptual basis for interpreting how alterations in microglial function could contribute to neurodevelopmental conditions such as ASD.

Future studies will be required to determine how mitochondrial transfer integrates with other microglial signals across diverse genetic backgrounds and developmental stages. Important next steps include defining the molecular pathways that enable mitochondria‐mediated maturation, assessing long‐term functional and transcriptomic consequences of microglial mitochondrial input, and extending these findings from in vitro systems to in vivo models. Such efforts will help clarify how microglial regulation of neuronal metabolism and development shapes human neurodevelopment and its disruption in disease.

## 5 Limitations

Our study uses a limited number of genetic backgrounds and an in vitro system, constraining generalization to ASD heterogeneity. The ASD comparison leverages available cortical datasets that may differ from our in vitro system in region, age, and cell‐type composition and was performed at a single early time point (48 h), limiting causal inference. RNA‐seq was conducted only at 48 h, preventing assessment of later transcriptional changes (for example, 1 week). In addition, the study does not include a genetic microglial mitochondrial loss‐of‐function model, and the uptake‐inhibition experiments used here represent functional rather than genetic perturbations of organelle transfer. Although the conditioned‐media and transfer‐inhibition experiments help separate the contributions of secreted factors from mitochondrial transfer, it is likely that additional microglial signals also influence the full maturation response. Future work incorporating multiple donor lines, extended time courses, in vivo validation, and targeted perturbations (for example, TLR2/TREM2 or integrin–ECM nodes) will help determine how maturation benefits can be preserved while minimizing ASD‐like immune convergence.

## 6 Methods

### 6.1 3D Silk Scaffold Model Preparation

#### 6.1.1 Silk Processing and Scaffold Fabrication

Silk scaffolds were prepared from silk fibroin following a previously established protocol [31, 32, 52]. Silk cocoons were boiled in a deionized water sodium carbonate solution (0.02 M) for 30 min to remove sericin. The silk fibroin fibers were dried overnight in a fume hood. The next day, the silk fibers were dissolved in 9.3 M lithium bromide (Sigma, cat. no. 213225) by immersion for 4 h at 60°C. Following the dissolving of the silk, dialysis of the silk in deionized water at room temperature was performed for 3 days in dialysis tubing to remove the lithium bromide from the silk (total of 6 water changes).

The resulting silk solution was centrifuged twice at 9000 rpm for 20 min, and a 100 µm strainer was used to filter out the remaining debris. Then, 500 µL of the silk solution was dried in a small weight boat at 60°C overnight to determine the weight/volume (w/v) concentration. The silk solution concentration was adjusted to 6 mg mL^−1^ and combined in a 10 cm dish with 400 to 500 µm sodium chloride (Sigma, cat. no. 71382) at a ratio of 1:2 (v/w). The silk solution was then left for 2 days at room temperature to trigger pore formation of the 3D silk sponge; the solution was then incubated for one hour at 60°C. After separating the sponge from the dish, residual sodium chloride was removed by dialysis in deionized water for 2 days at room temperature with 6 water changes total (3 times a day).

To create the scaffolds for experimental purposes, the sponges were cut into individual donut‐shaped scaffolds using biopsy punches (Integra) with a 6 mm OD (outer diameter) and 2 mm ID (inner diameter). The scaffolds were trimmed to a height of 1.5 mm using scissors. Following the shaping of the scaffolds, they were then autoclaved in deionized water for 20 min with the liquid cycle, cooled to room temperature, and then processed for experiments (process described below).

### 6.2 Coating With ECM

Extracellular matrix coating of scaffolds was done using previously generated protocols [32]. In brief, scaffolds were coated with 10 µg mL^−1^ poly‐ornithine (PLO) (Sigma, cat. No. P4957) and 5 µg mL^−1^ laminin (Fisher, cat. no. 501003381) to allow for proper cell adhesion. Scaffolds were placed into a 6‐well plate (maximum of 50 per well) using tweezers and incubated overnight at 37°C in 7 ml of a 10 ug mL^−1^ PLO coating solution in distilled water. The next day, the PLO was aspirated and washed three times with PBS. The scaffolds were then incubated overnight at 4°C in 0.5 mg mL^−1^ 100 µL laminin stock aliquots with Dulbecco's Modified Eagle Medium/Nutrient Mixture (DMEM F12) phenol red‐free media in a 1:100 ratio and then the scaffolds were retained in the laminin solution. Before use, the scaffolds were rinsed with PBS, followed by adding 5 mL of media of interest.

### 6.3 Cell Culture

#### 6.3.1 Fluorescent Mitochondria Cell Lines (mtDsRed2 hiNSCs and mtEBFP2 Microglia)

We used previously generated in our lab mt‐dsRED2 hiNSCs and mt‐BFP2 microglia following the established protocol [53]. In brief, hiNSCs and HMC3 human microglia cells were transduced with hSYN‐mito‐dsRED2 plasmid (Addgene, 173069) for hiNSCs and a custom plasmid used VectorBuilder to implement a double COX8A promoter and EBFP2 into the plasmid backbone for HMC3s [33, 41].

### 6.4 Mouse Embryonic Fibroblasts (MEFs)

Following ATCC protocols, mouse embryonic fibroblasts (MEFs) (ATCC: SCRC‐1008, Mycoplasma contamination testing completed by vendor, the new vial of cells was used after passage 5) were used as a feeder layer for induced human neuronal stem cells (ihNSCs). The cells were seeded on a 15 cm^2^ gelatin‐coated dish (0.1% gelatin for 20 min: Sigma–Aldrich SF008). MEFs were used at passages 3–4. DMEM supplemented with 10% Fetal Bovine Serum (FBS), and 1% Antibiotic‐antimycotic (Anti‐Anti) were used to maintain the MEFs. The media was changed every 3 days.

Once the MEFs reach 100% confluency, they are then inactivated with 10 µg mL^−1^ Mitomycin C (Sigma, M4287) for 3 h, followed by three washes with Phosphate Buffered Saline (PBS). After the last PBS wash, media was added to the plates, for hiNSC seeding.

### 6.5 mt‐DsRed2 Induced Human Neural Stem Cells (hiNSCs)

mt‐dsRED2 hiNSCs were generated from healthy human male neonatal foreskin fibroblasts and maintained following previously established protocols [31, 32]. The cell line was checked for mycoplasma before starting. The media was composed of KnockOut DMEM supplemented with 1% GlutaMax, 20% KnockOut Serum Replacement, 1% Anti‐Anti, 0.2% β‐mercaptoethanol (Invitrogen), and 800 µL of 10 µg mL^−1^ basic fibroblast growth factor (Invitrogen) added proportionally to aliquots of media upon use. Media was changed every other day. mt‐dsRED2 hiNSCs were expanded and cultured on top of inactivated mouse embryonic fibroblasts. When cells reached 70–80% confluency, they were lifted from the plates via incubation with TrypLE solution for 1 min at 37°C, followed by inactivation with mt‐dsRED2 hiNSCs media. Cells were then pelleted by centrifugation at 3000 rpm for 2 min. For further expansion, pellets were gently mechanically disrupted with a 5 mL pipette, and the colony suspension was transferred to a Matrigel‐coated dish (1:50 dilution of Matrigel in phenol red‐free DMEM F12 for 1 h). mt‐dsRED2 hiNSCs colonies were replated into the Matrigel‐coated dishes following a 1:20 ratio of 15 cm^2^ dishes. For 3D seeding, the pellet was rigorously pipetted to obtain single‐cell suspension along with centrifugation at 1000 rpm for 5 min.

### 6.6 mt‐EBFP2 HMC3 Microglia Cell Line

The transduced mt‐EBFP2 HMC3 microglia cell line (ATCC: CRL‐3304, contamination testing completed by vendor) was maintained following established ATCC protocols [33]. Fluorescent mitochondria microglia were expanded in 15 cm^2^ dishes in EMEM media supplemented with 10% FBS, and 1% Anti‐Anti. The media was changed every 3 days. When cells reached 70%–80% confluency, they were lifted from plates with 0.25% Trypsin/EDTA for 3 min at 37°C. After adding complete media to the mt‐EBFP2 microglia, cells were pelleted with centrifugation at 1000 rpm for 5 min. Cells were replated at 300 000 cells cm^2^.

### 6.7 3D In Vitro Human Brain Tissue Model Fabrication

The system for generating 3D human in vitro brain tissue model was done via an earlier published protocol [31, 32]. Once mt‐DsRed2 hiNSCs and mt‐EBFP2 HMC3s reached nearly 100% confluency, they were collected as described in the individual culture methods and combined to achieve the following ratio: 2:0.1 million neurons and microglia, respectively. The same ratio was used for monocultures for the respective cell types. Immediately before seeding the mono‐ or co‐culture cell solution, PLO‐ and laminin‐coated silk scaffolds were placed in 96‐well plates with tweezers and an aspirator line to remove excess liquid. Calculations were performed so that each 40 µL of cell suspension contained 2:0.1 million mt‐DsRed2 neurons and mt‐EBFP2 microglia. Then 40 µL of the cell suspension was pipetted onto the semi‐dried scaffolds and incubated for 30 min at 37°C to allow the cells to attach. After this, 150 µL of complete neurobasal media was added to each well and then placed in an incubator at 37°C with 5% CO_2_ in a humidified atmosphere for 24 h to allow cells to sufficiently attach to silk scaffolds. Complete neurobasal media (CNB) was composed of Neurobasal medium supplemented with 2% B‐27, 1% Anti‐Anti, 1% Glutamax, and 1% astrocyte growth factors.

The following day, the cell‐seeded scaffolds were transferred to new 96‐well plates using sterile tweezers. To ensure a full 3D environment, each scaffold was embedded in100 µL of collagen type I solution with complete neurobasal media, 10x PBS, and a pH adjusted to 7.0–7.2 with NaOH and then incubated for 30 min at 37°C to allow the collagen gel to crosslink. Next, 150 µL of complete neurobasal media was added to all scaffolds and incubated for 24 h at 37°C. The next day, the brain‐like tissues were moved into 48‐well plates with 1 ml of complete neurobasal media in each well. Half of the media changes were done every fourth day until the tissues were mature enough for experimental analysis (dense neuronal network, and spontaneous neuronal activity or 5–6 weeks after seeding [53].

### 6.8 Mitochondria Isolation, Treatment, and Analysis

#### 6.8.1 Microglia Mitochondria Isolation and Treatment of 3D In Vitro Brain Cultures

*Intracellular Isolation*: Mitochondria isolation was conducted using the established protocol from the Thermofisher Scientific Mitochondria Isolation Kit for Cultured Cells (Thermo Fisher: 89874). In brief, 6‐week old scaffolds were transferred into 2 mL Eppendorf tubes and serial treated with supplemented reagents A–C, followed by mechanical breakage of the scaffold, and 5 min max speed vortexing. Next, the large pieces of silk debris were removed from the Eppendorf tubes, followed by removal of small ECM, cell, and silk debris after 10 min centrifugation at 1000 × g (4°C). The mitochondria fraction was pelleted by 25 min of centrifugation at 12 000 × g (4°C). Isolated mitochondria were either used for treatment or analysis. The residual protein‐rich supernatant was stored in 2 mL Eppendorf tubes at −80°C for Western Blot analysis.

*Extracellular Isolation*: The media from 3D in vitro cultures was collected into the 1.5 mL Eppendorf tubes and centrifuged for 10 min at 1000 × g to pellet the cells. The supernatant was centrifuged at 12 000 × g for 25 min to collect a mitochondria‐enriched fraction. The residual mitochondria‐free media after centrifugation was stored at −80°C for later analysis.

### 6.9 Treatment of Naïve Neurons

Mitochondria‐enriched CNB—immediately before the treatment, the pelleted microglia mitochondria were reconstituted in 1 mL of CNB media, and this solution was then used for the treatment protocol. In brief, to deliver the mitochondria‐enriched CNB, the entire media volume was removed from the naïve neuronal cultures (48‐well plate – 1 mL) and replaced with 1 mL of mitochondria‐enriched CNB for the “Treated” conditions. “Untreated” and “NM” conditions also received a full media change to maintain consistency within the experiment.

### 6.10 Microglia Conditioned Media Preparation and Neuronal Treatment

HMC3 human microglia were cultured in 3D silk scaffolds in Neurobasal media supplemented with B27, GlutaMAX, and AGS (NB+AGS) and matured for five weeks under previously established conditions [32]. At week 5, conditioned media from cultures with at least 3 days of culture were collected by centrifugation at 1000 × g for 10 min to remove residual cells and debris, followed by centrifugation at 12 000 × g for 15 min to remove mitochondria. The resulting mitochondria‐depleted microglia‐conditioned media were immediately transferred to neuronal cultures. For 48 h experiments, conditioned media fully replaced the neuronal culture medium. For 1‐week experiments, media were refreshed every 72 h to maintain consistent exposure to microglial secreted factors. Neurons maintained in NB+AGS without microglial exposure served as controls.

### 6.11 Pharmacological Inhibition of Microglia‐Neuron Mitochondrial Transfer

To block mitochondrial transfer in neuron‐microglia co‐cultures (NM), two mechanistically distinct inhibitors were applied: Rasarfin, an endocytic trafficking inhibitor, and Ciliobrevin D, a dynein ATPase inhibitor [54, 55]. Both compounds were used at concentrations previously reported to impair organelle trafficking without inducing overt cytotoxicity (Rasarfin: 10 mM; Ciliobrevin D: 10 mM). NM co‐cultures received the first inhibitor treatment at day 14 post‐seeding, coinciding with the onset of spontaneous mitochondrial transfer. Drugs were refreshed every 72 h for a total of three weeks, matching the duration of the experiments in this study. DMSO‐treated NM cultures served as controls. Cell viability and morphology were monitored throughout the treatment period to ensure that observed phenotypes were not attributable to toxicity.

### 6.12 Mitochondria Bioenergetic Analysis via Seahorse Assay

The mitochondria's bioenergetic function was measured using Seahorse Real‐Time ATP Rate Assay following the manufacturer's instructions with minor optimizations for our system. In brief, 12–18 h prior to running the Seahorse Real‐Time ATP Rate Assay, the sensors from the Extracellular Flux Pack (Agilent Technologies: 103792‐100) were hydrated with 200 µL of sterile water the day before the assay and stored in a 37°C non‐CO_2_ incubator overnight. The day of the assay, the sterile water was removed and 200 µL of Seahorse XF Calibrant was added to the utility plate for at least 2–4 h. The assay medium was prepared with 50 mL of phenol‐red free DMEM F12, 10 mM of XF glucose, 1 mM of XF pyruvate, and 2 mM of XF glutamine. Stock compounds were made on the day of the assay with resulting stock concentrations of 150 µM for Oligomycin and 50 µM for Rotenone + Antimycin A. These stock solutions were then diluted in prepared media (1:10).

Following the preparation of assay compounds and Seahorse media, mitochondria were isolated from the 3D silk scaffolds following the procedure above at select time points (24 and 48 h, and 1‐week post‐treatment). Once the mitochondria (intracellular and extracellular) were isolated, they were then reconstituted in 180 µL of Seahorse media and added to the Cell Culture Microplate. All empty wells were filled with 180 µL of Seahorse media to be used as a negative control and blanks for the assay. Using the established protocol from Agilent Technologies for the Standard Assay, 20 µL of the final prepared Oligomycin is added into Port A and 22 µL of Rotenone + Antimycin A into Port B of the Sensor Cartridge.

After the loading of the mitochondria and assay compounds, the mitochondria were analyzed via the Seahorse XFe96 Analyzer using the “Real‐Time ATP Rate Assay” template to measure Oxygen Consumption Rate (OCR) and Extracellular Acidification Rate (ECAR). Data collected was used for further analysis of mitochondrial function. Succeeding the Real‐Time ATP Rate Assay, 20 µL of the mitochondria‐rich media was stored at −20°C for mtDNA quantification. The remaining 160 µL is centrifuged at 12 000 × g for 20 min and reconstituted in 60 µL of RIPA (supplemented with protease and phosphatase inhibitors) buffer for storage and later analysis.

### 6.13 Mitochondria Transfer and Progression of Neuronal Maturation

#### 6.13.1 Immunofluorescence Staining and Analysis

PBS solution supplemented with 4% sucrose and 4% paraformaldehyde (PFA, Electron Microscopy Sciences), was used to fix the scaffolds at the selected (indicated) timepoints. After fixing, the tissues were washed four times with PBS and then permeabilized for 1 h with a permeabilization solution composed of 0.2% Triton X‐100 and 4% goat serum. Using the permeabilization solution, primary antibodies were diluted (anti‐Tuj1: ab78078 mouse; 1:1000) and added to the scaffolds. The scaffolds were incubated in the primary antibody at 4°C overnight on a rocker, then washed with gentle shaking in PBS four times (10 min each wash). After washing, tissues were incubated on a shaker with secondary antibodies diluted in PBS for 1 h at room temperature (goat anti‐mouse Alexa 647: A11126; 1:500). Four additional PBS washes were used to dispose of any unbound antibodies. For all figures, fluorescent image stacks of the stained scaffolds were acquired on a Nikon A1 inverted LUNV confocal microscope (Nikon, Plan Apo λ 20x, 1024 × 1024, each maximum projection had 50 steps). Images in the figures represented maximum intensity projection and were collected with the same PMT gain settings and laser power between all experiments. All scaffolds were imaged with the EBFP2 line blind to avoid bias. Tuj1 neuronal network density was analyzed using a custom MATLAB code [31].

### 6.14 Cell‐Specific Mitochondrial Aspect Ratio Analysis

In brief, the ratio of mitochondria length versus width (aspect ratio) was measured using a publicly available macro from NIH ImageJ software [56]. Max projection images of fluorescent mitochondria acquired with Nikon A1 inverted LUNV confocal microscope (1024 × 1024 pixels, 20x objective) were analyzed via the macro. The measured aspect ratio for each mitochondrion was plotted as a distribution graph for comparison with other groups.

### 6.15 Mitochondrial DNA Quantification

Using previously established protocols with minor optimizations for our system [Invitrogen: P11496]. Previously prepared samples from the Seahorse Assay were thawed on a shaker and buffer solutions were prepared (1X TE buffer and 0.2% Triton X). While waiting for samples to thaw, standards for the standard curve were prepared in a 48‐well plate with 1X TE buffer and DNA working solution (working solution: 1:50 dilution of DNA Lambda in 1X TE buffer). The final concentrations for the standards are as follows, 2000, 1000, 500, 200, 100, 50, 20, and 0 ng mL^−1^. Once the samples are thawed, dilute your sample with 1X TE buffer and 0.2% Triton X (1:5 dilution of sample) for a total sample volume of 100 µL. Once the samples are prepared, they are transferred to a black 96‐well plate. The PicoGreen solution was prepared so there was 100 µL per sample and standard (1:200 dilution in 1X TE buffer).

Prior to the addition of the PicoGreen Solution, the plate reader was set to fluorescent excitation = 485 nm, emission = 538, and shake for 5 s. Once the plate reader is set up. In a dim room, add 100 µL of PicoGreen Solution to the wells with samples and standards, then read the plate, continuing reading until the reaction is completed. For analysis, a standard curve was generated from the standards on the plate. Followed by dilutions and application of standard equation to generate mtDNA content per sample.

### 6.16 Mitochondria Colocalization Analysis

Colocalization analysis was completed using the JACoP plugin from the NIH ImageJ software [57]. Images were acquired with Nikon A1 inverted LUNV confocal microscope (1024 × 1024 pixels, 20x objective) were analyzed via the macro with the Otsu thresholding method. Spearman's correlation coefficients were plotted by condition for comparison with other groups and/or timepoints. For each image stack, colocalization between mtDsRed2‐positive neuronal mitochondria and mtEBFP‐positive microglial mitochondria was quantified across all 50 individual z‐slices rather than on a single projection.

### 6.17 Western Blot

For whole scaffolds, 1X RIPA lysis buffer (with protease and phosphatase inhibitors) was added to extract cellular protein lysates. Scaffolds were subsequently sonicated at 20% amplitude for 20 pulses (1 s pulse on, followed by 1 s off). Sonicated samples were then diluted at a 3:1 ratio with a sample buffer (9:1 Laemmli buffer to β‐mercaptoethanol). Samples containing isolated mitochondria (intracellular and/or extracellular) or total protein fraction were obtained during the Seahorse Assay and post‐processing. For analysis, 15 µL of the sample was mixed with 5 µL of the sample buffer (15‐well gel), while post‐Seahorse Assay protein samples were prepared with 45 µL of the sample and with 15 µL of the sample buffer (10‐well gel). Prepared samples were heated at 95°C for 5 min prior to loading onto pre‐cast gels for electrophoresis. Electrophoresis was conducted at 150 V for approximately 50 min to 1 h. Gels were imaged using the Stain‐Free preset on a Bio‐Rad imager to obtain total protein volume per lane.

If sample numbers exceeded the well capacity of a single gel, samples were split across two gels. To ensure consistency in detection, gels were sectioned at the same molecular weight markers (75 and 20 kDa) and transferred onto a polyvinylidene fluoride (PVDF) membrane. Protein transfer from gels to PVDF membranes was performed using a semi‐dry transfer system at 2.5 V and 1.6 A for 4 min (for total protein samples) or 3 min (for mitochondrial samples). Membranes were imaged using stain‐free blot imaging to assess total protein content, facilitating subsequent normalization.

Membranes were blocked for 5 min using Everything Blocking Buffer (Bio‐Rad, 12010020) and then were incubated with primary antibodies diluted in Everything Blocking Buffer at 4°C overnight (primaries listed in Table 1) overnight at 4°C on a rocker. After primary incubation, membranes were washed with 1X Tris‐Buffered Saline with Tween 20 (TBST) (2 instant washes, followed by 3, 10 min washes) to remove excess primary antibody. Membranes were then incubated with secondary antibodies (1:10 000 dilution in Blocking Buffer, Cell Signal Technologies, HRP—conjugated: anti‐rabbit IgG 7074P2; anti‐mouse IgG 7076S) for 1 h at 4°C, followed by the same wash sequence. For signal detection, membranes were exposed to an ultra‐sensitive enhanced chemiluminescent (ECL) substrate for 5 min before chemiluminescence imaging. Following imaging, membranes were stripped with a western blot stripping buffer at room temperature for 30 min, followed by the same TBST wash sequence, to enable repeated antibody staining for multiple target proteins. The images were analyzed using ImageJ Software.

**TABLE 1:** Primary Antibodies.

Primary Antibody	Animal	Dilution	Product Code
Anti‐pDRP1	Rabbit	1:1000	Cell Signaling – 3455S
Anti‐DNM1L	Mouse	1:1000	Invitrogen—MA5‐26255
Anti‐ Synapsin 1	Rabbit	1:1000	Abcam—AB64581
Anti‐MAP2	Rabbit	1:1000	Abcam—AB32454
Anti‐CX3CL1	Mouse	1:1000	Invitrogen—MA1‐031
Anti‐MTCO1	Mouse	1:1000	Invitrogen – 459600
Anti‐VDAC1	Rabbit	1:1000	Cell Signaling – 4661S
Anti‐Tuj1	Mouse	1:1000	Abcam—AB78078
Anti‐TOMM20	Rabbit	1:1000	Invitrogen—MA5‐24859

### 6.18 RNA Isolation Protocol

Scaffolds were collected and stored at −80°C. Upon isolation, samples were thawed on ice and incubated with 600 µL of Lysis Buffer from RNeasy Kit (Qiagen, Catalog No. 74106). During incubation, samples were cut up using small scissors and sonicated to break up the scaffold (Amplitude – 20%, Time – 5 s, 1 s on 1 s off). After homogenization, the samples continued to sit in a lysis buffer on ice for approximately 20 min. The lysed samples were placed onto QIAshredder Mini Spin (Qiagen) columns to remove pieces of silk and spun at 15 000 rpm for 2 min. An equal amount of 70% ethanol was added to the supernatant and mixed thoroughly. The ethanol‐containing mixture was spun in two rounds through the RNeasy Mini Spin column (Qiagen) at 10 000 rpm for 1 min each; the flow through was discarded. In a new collection tube, 350 µL of RNA wash from RNeasy Mini Kit (Qiagen) added to the spin column and incubated for 5 min. The sample was added to the RNeasy column and spun at the same setting for 1 min; the flow through was discarded. After the first RNA Wash, TURBO DNase treatment was prepared and completed following manufacturer's instructions (Invitrogen, cat. no. P5537155). After the final spin at 10 000 × g for 1.5 min, a second and third round of RNA was performed as well as a final spin to ensure the column was dry. The column was placed into a 1.5 mL microcentrifuge tube, and 24 µL of RNase‐free water (Fisher Scientific) was added to the center of the column, incubated for 10 min, and spun at 10 000 rpm for 1 min (two times). Samples were then read on the Tecan Infinite M Plex NanoQuant Plate and stored at −80°C before sequencing.

Azenta Life Sciences completed sequencing, data was analyzed using Python comparing NM to Treated and Treated to Untreated conditions (*p*‐value < 0.05, log2(fold change) = 0.2). Differentially expressed genes were then compared to the MitoCarta 3.0 to delve into potential bioenergetic pathways [34]. Heatmaps and volcano plots were generated with matplotlib Python package.

### 6.19 RNA Sequencing Analysis

Raw counts were obtained from GEO Bioproject PRJNA268211 in Supporting files (GSE64018_countlevel_12asd_12ctl.txt.gz) [35] and normalized using DESeq2. To compare our in vitro data with human data, Ensemble IDs were first converted to HGNC symbols using biomaRt. DESeq2 was then used on ASD vs CTL groups to obtain DEGs with p.adj < 0.05. These DEGs served as the autism baseline for comparison with this model. It was next performed DESeq2 on NM, NTr, and NUntr and carried out serial contrasts between these groups (NM vs NTr, NM vs NUntr, and NTr vs NUntr). Using the DEGs identified in the ASD vs CTL groups, the same genes in this serial contrasts was subset. By comparing log2FoldChange and p.adj values, the degree of convergence and divergence of this model relative to human data was determined.

To evaluate similarities and differences between this model and human datasets, it was subset DEGs from all contrasts that were either convergent or divergent with ASD vs CTL. ClusterProfiler was used to convert HGNC symbols to Entrez IDs, and ReactomePA was applied to perform over‐representation analysis. Pathway‐level results were visualized by generating a heatmap of genes grouped by shared pathways and a lollipop plot of divergent Reactome pathways.

To further dissect differences between groups, it was focused on the subset of DEGs (derived from ASD vs CTL DEGs) and excluded genes that overlapped with the “all convergent” and “all divergent” datasets. These remaining DEGs were analyzed using ClusterProfiler and ReactomePA for over‐representation analysis, and lollipop plots were created for both convergent and divergent pathways. Finally, to specifically assess ASD‐associated biology, an ASD gene pathway dataset by combining ASD‐related genes from BioGRID, SFARI Gene, and AutDB was curated. ReactomePA was used to perform over‐representation analysis, and pathways with p.adjust < 0.05 were retained. Gene set enrichment analysis (GSEA) was then performed on the normalized counts using this curated ASD gene pathway dataset.

### 6.20 Statistical Analysis

Statistical analysis was performed between and within experimental groups using GraphPad Prism software. Two‐tailed t‐tests were used to compare values within an experimental group and between two experimental groups. Mann‐Whitney U and Welch's t‐tests were used when data did not meet normality and variance assumptions. One‐ or two‐way ANOVA analysis of variance was used to compare multiple groups. Tukey's or Dunnett's post hoc tests were used to assess computed significant differences between experimental and control groups when the data was normal. Kruskal‐Wallis was used with Dunn's post‐hoc test when data was nonparametric. Repeated measure ANOVA was used to compare control and experimental conditions, when necessary, followed by Dunnett's post‐hoc test. Any *p*‐value less than 0.05 was considered statistically significant. Each experiment was repeated at least two times; technical replicates (2–3, depending on the experiment) were used for every assay. Data was represented by mean and standard error of the mean (SEM) of each group. Outlier elimination was done with Grubbs’ or ROUT. Power analysis was completed at a power of 80% with an α value of 0.05, leading to *n* = 3 for sham and *n* = 4 for experimental condition. Independent experiments were run in duplicate or triplicate.

## Author Contributions

**V.L**. and **S.P.S**. conceived the project. **V.L**. and **S.P.S**. designed and interpreted the experiments and wrote the manuscript. SBSK fabricated and provided fluorescent mitochondria cell lines. **S.P.S**. and **V.L**. performed scaffold seeding and sample maintenance (media changes). **S.P.S**. performed all mitochondrial treatments. **S.P.S**. and **V.L**. performed mitochondrial isolation. **S.P.S**. performed all assessments of mitochondrial bioenergetic function. **V.L**. performed confocal imaging. **S.P.S**. and **C.C.A**. conducted Western Blots. **S.P.S**. performed aspect ratio, mtDNA quantification, colocalization, and neuronal network analysis. **S.P.S**. prepared silk materials. **S.P.S**. and **V.L**. conducted mRNA isolation. **S.P.S**., **S.B.S.K**., and **V.K**. performed **R.N.A**. sequencing analysis. **C.C.A**. performed live mitochondria movement imaging. **C.C.A**. performed microglia media treatment and mitochondrial transfer inhibition experiments, and WB staining; **V.L**. analyzed these experiments.

## Conflicts of Interest

The authors declare no conflicts of interest.

## Supporting information




**Supporting File 1**: advs74505‐sup‐0001‐SuppMat.docx.

**Supporting File 2**: advs74505‐sup‐0002‐VideoS1.mov.

**Supporting File 3**: advs74505‐sup‐0003‐Data.zip.

## Data Availability

All data generated or analyzed during this study are included in this article (and its supporting information files). This study's mRNA sequencing data set was deposited in the GEO repository database (https://www.ncbi.nlm.nih.gov/geo/) with accession number GSE319314.
