# Supplementary material for: Microglia Mitochondria Support Neuronal Maturation via Metabolic and Transcriptional Reprogramming in Human 3D In Vitro Brain Model
Source: Adv Sci (Weinh). 2026 Mar 13;13(25):e08815. doi: 10.1002/advs.202508815 (PMC13137835; doi:10.1002/advs.202508815)
Supplement: Supplementary file 1 — Supporting File 1: advs74505‐sup‐0001‐SuppMat.docx. [file ADVS-13-e08815-s003.docx]

**Title: Microglia Mitochondria Support Neuronal Maturation via Metabolic and Transcriptional Reprogramming in Human 3D In Vitro Brain Model**

^1^ Sydney P. Sterben, ^1^ Charitha C Anamala, ^1^ Sahan B.S. Kansakar, ^1^ Vaishnavi Koduri, ^1,2^ Volha Liaudanskaya

^1^ Department of Biomedical Engineering, University of Cincinnati, Cincinnati, OH, USA

^2^ Neuroscience Graduate Program, University of Cincinnati, College of Medicine, Cincinnati, OH USA

Corresponding Author: Volha Liaudanskaya, 3159 Eden Avenue, Cincinnati, OH, USA; [liaudava@ucmail.uc.edu](mailto:liaudava@ucmail.uc.edu)


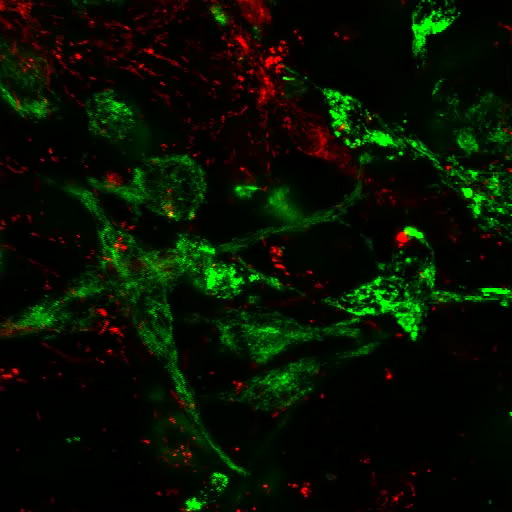
Supplementary Information

*SVideo 1: Time-lapse imaging of neuron–microglia co-cultures over 6 hours (1 frame/2 min). Red represents neuronal mitochondria and green represents microglial mitochondria. Video was recorded with 20x objective and 2x optical zoom. The video highlights dynamic interactions and transfer events between neuronal and microglial mitochondria. Scale bar, 10 μm.*


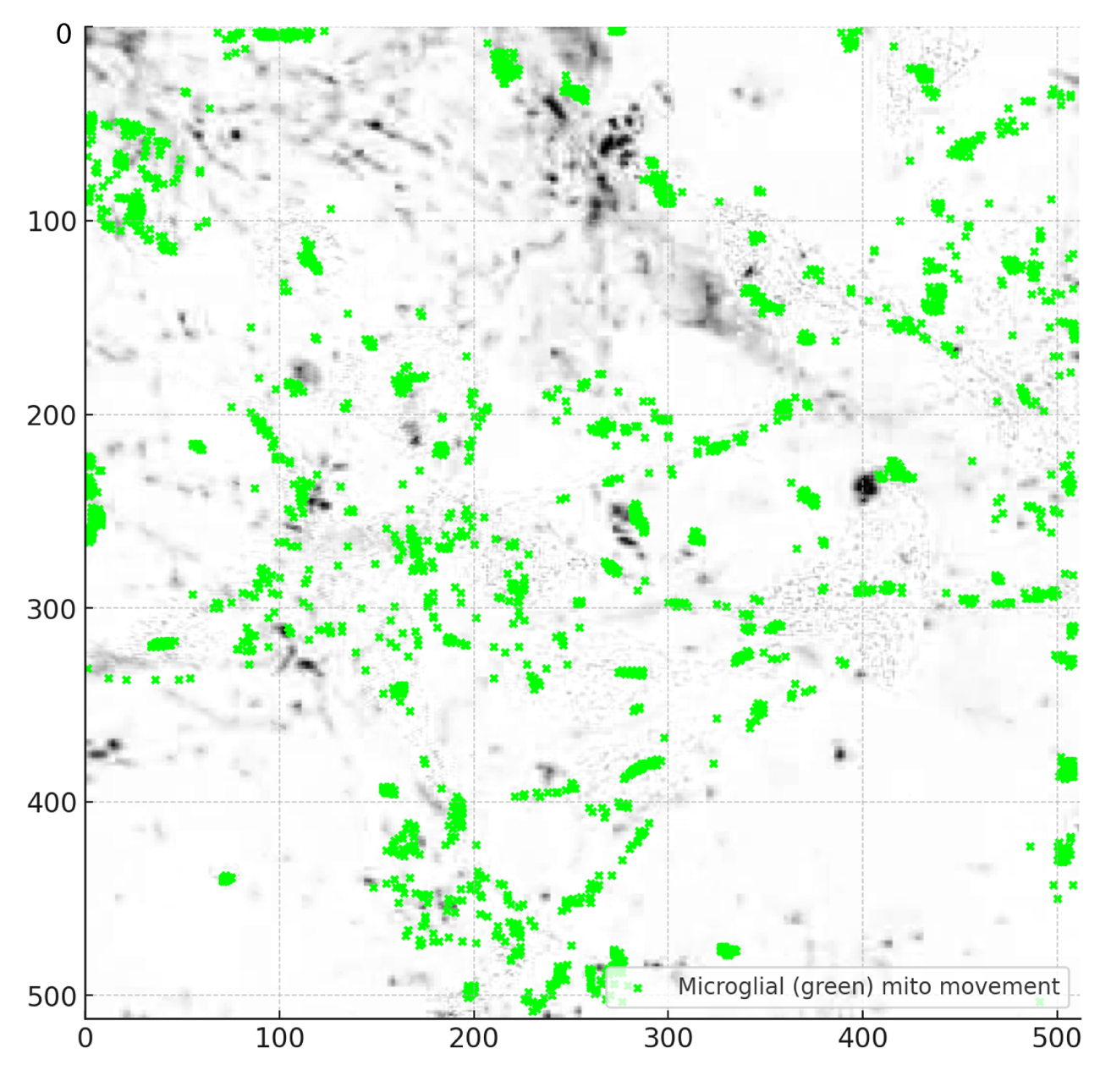


*Figure S1: Tracing of microglial mitochondria movement. Neuronal mitochondria (black) overlaid with tracked microglial mitochondrial movements (green dots). The image was generated by isolating the neuronal mitochondrial channel and mapping positions of migrating microglial mitochondria across the time-lapse sequence, highlighting the spatial overlap between neuronal structures and incoming microglial mitochondria.*


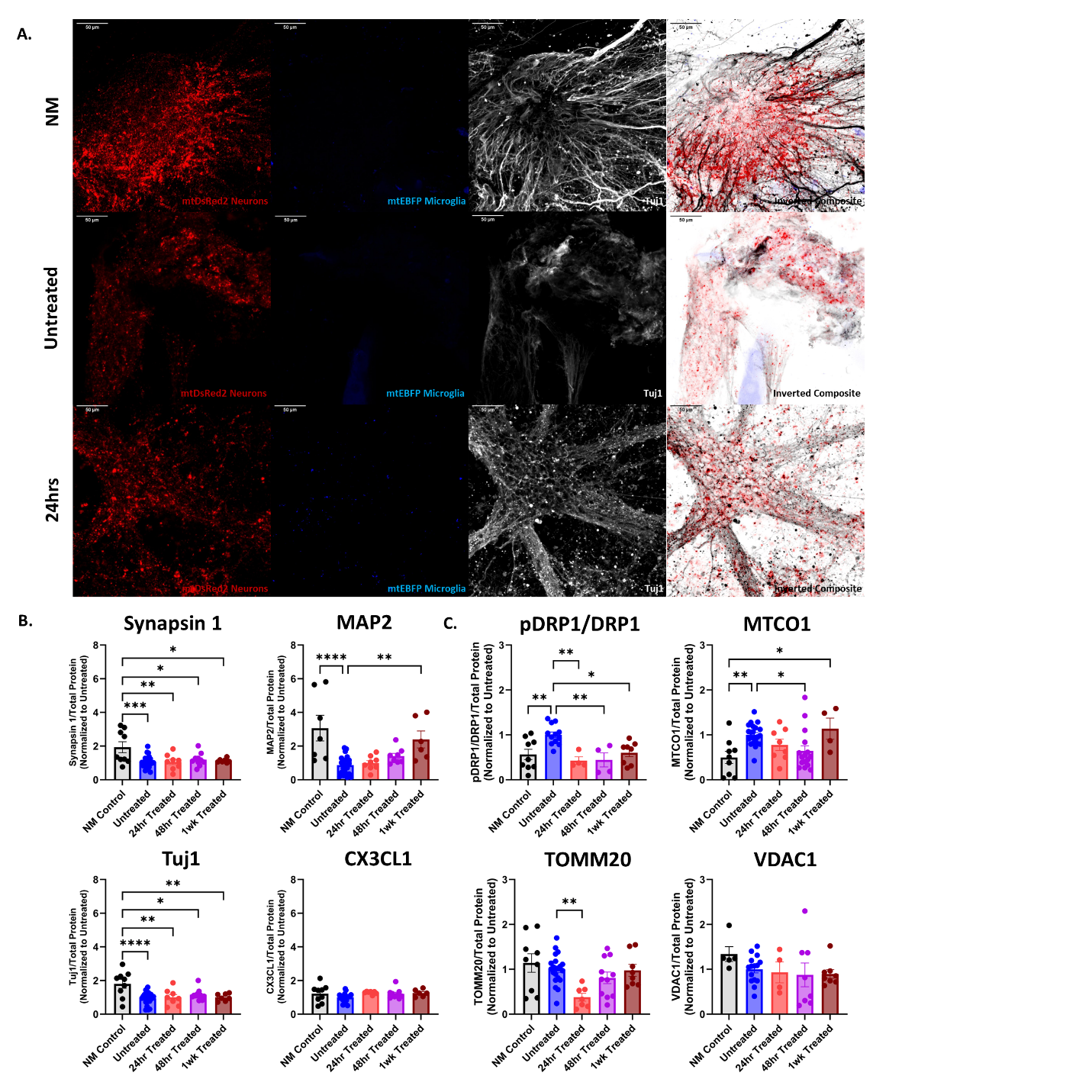


Figure S2: 24hr Treatment – Maturation and Mitochondrial Health: **A.** Representative images of NM, Untreated, and 24hrs Treated. mtDsRed2 neurons and mtEBFP2 microglia. Tuj1 staining for neuronal networks. Nikon A1 inverted LUNV confocal microscope 20x objective 1024x1024 pixels with 50-steps Max projection. Scale bar: 50µm. **B.** Neuronal maturation markers MAP2, CX3CL1, Synapsin 1, and Tuj1 over time western blots normalized to total protein concentration per lane and to Untreated condition. **C.** Mitochondria health markers, pDRP1/DRP1, TOMM20, VDAC1, and MTCO1 intracellular mitochondria western blots normalized to total protein concentration per lane and to Untreated condition. n = 9 for NM and Untreated. n = 12 for 24hr Treated. Mean ± SEM. One-way ANOVA with Tukey post-hoc test and α = 0.05. ROUT outlier analysis method, outliers were removed MAP2 (1wk Treated) and MTCO1 (1wk Treated). Analysis completed in GraphPad Prism. Each timepoint was replicated at least twice.


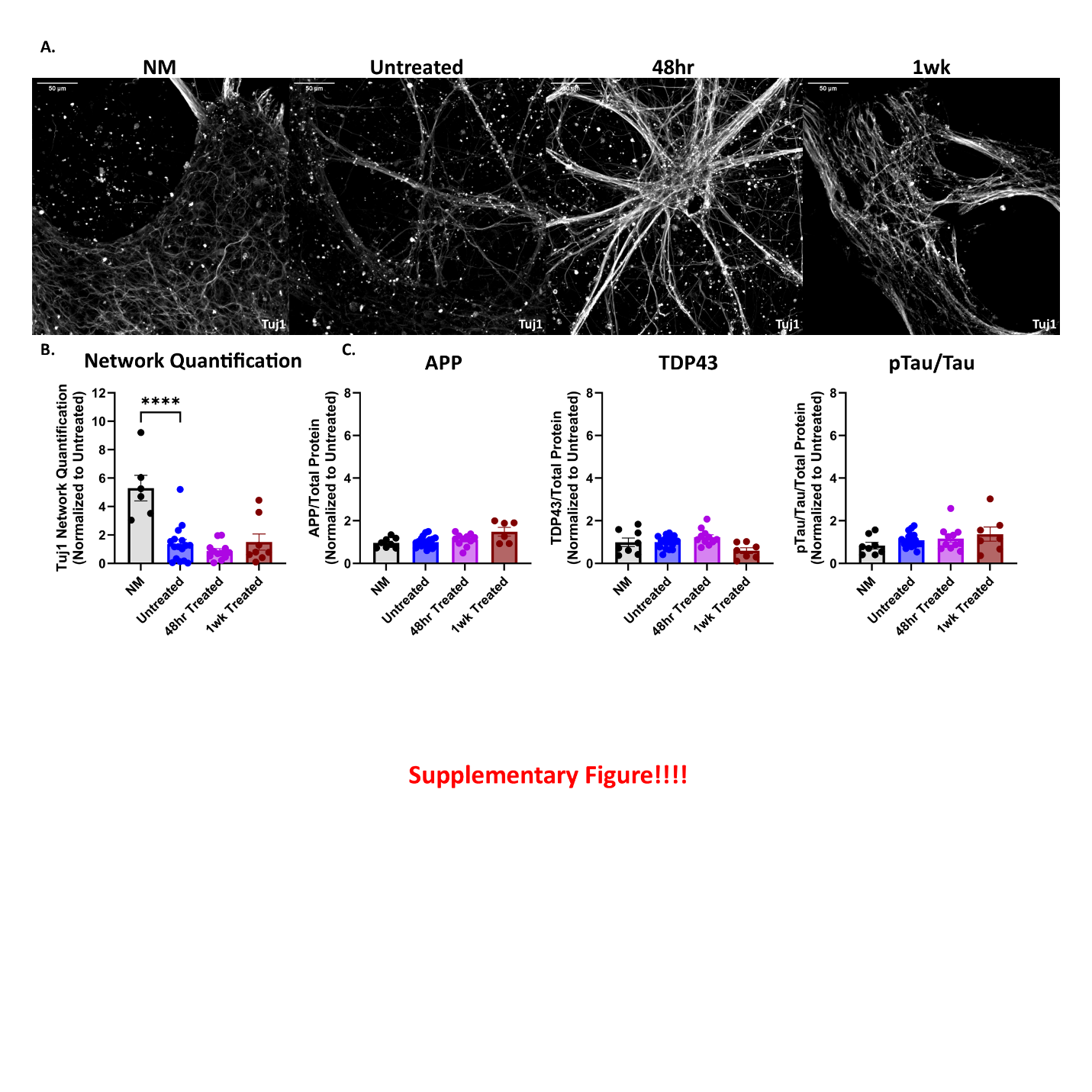


*Figure S3: Treatment and Neurodegeneration:* ***A.*** *Representative images of NM, Untreated, 48hrs, and 1wk Treated. Tuj1 staining for neuronal networks. Nikon A1 inverted LUNV confocal microscope 20x objective 1024x1024 pixels with 50-steps Max projection. Scale bar: 50µm.* ***B.*** *Neural network quantification with custom MATLAB code comparing NM, Untreated, 48hrs, and 1wk Treated* (45)*.* ***C.*** *Neurodegeneration markers, APP, TDP43, and pTau/Tau western blots normalized to total protein concentration per lane and to Untreated condition. n = 3 for NM and Untreated. n = 4 for 48hr and 1wk Treated. Mean ± SEM. One-way ANOVA with Tukey post-hoc test and α = 0.05. ROUT outlier analysis method, outliers were removed for Network Quantification (NM Control), APP (1wk Treated), TDP43 (1wk Treated), and pTau/Tau (1wk Treated). Analysis completed in GraphPad Prism. n = 9 for NM and Untreated and n = 12 for Treated. Each timepoint was replicated at least twice.*


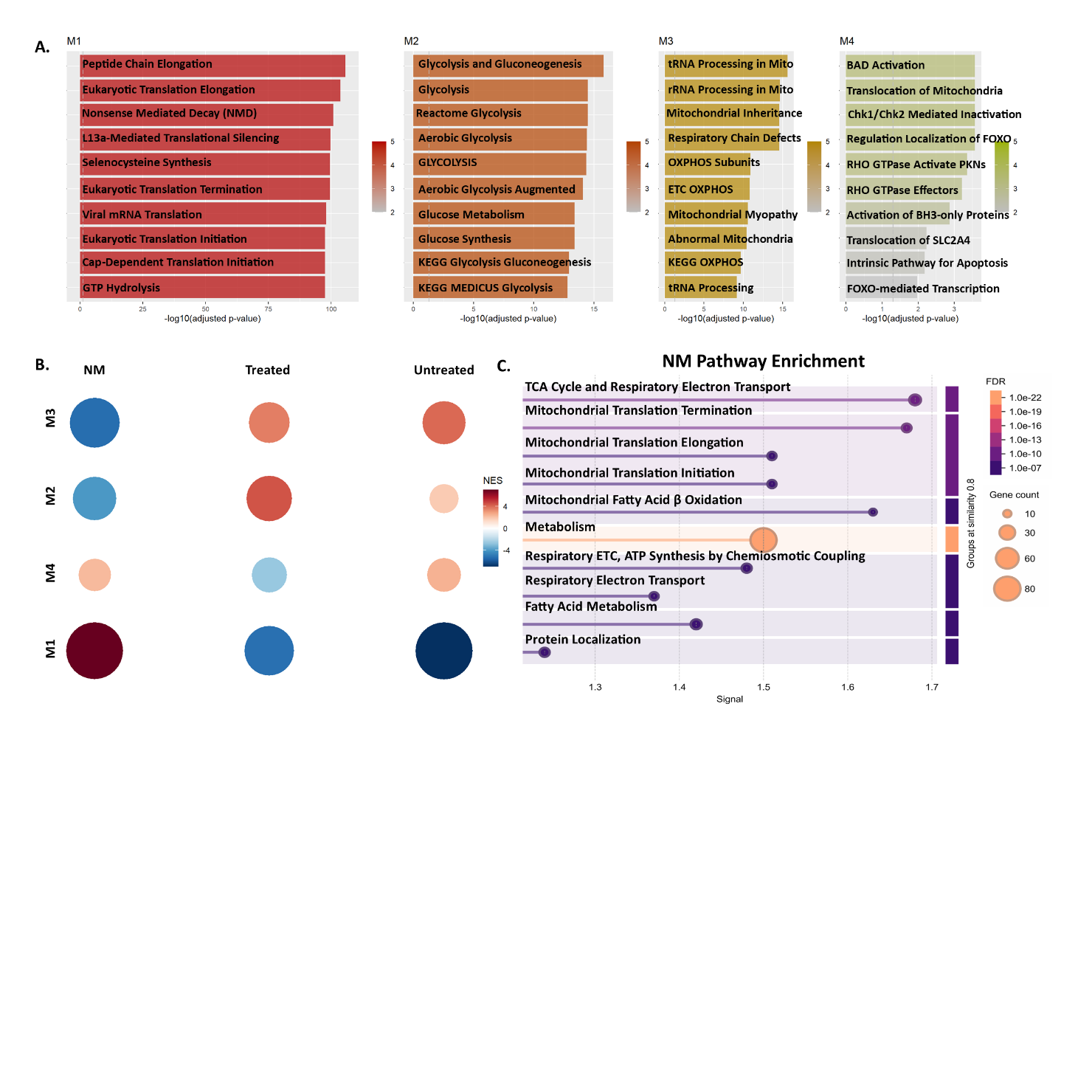


*Figure S4: CEMiTool Pathway Enrichment:* ***A & B.*** *Gene set enrichment analysis demonstrating 5 modules with M1 – Protein Production (positively associated with NM), M2 – Glycolysis (positively associated with Treated), M3 – OXPHOS and Mitochondria Dysfunction (negatively associated with NM, positive with Untreated), and M4 – Mitochondrial Motility (positively associated with Untreated).* ***C.*** *NM mitochondrial pathway enrichment genes from Mito Carta 3.0 in NM. Log_2_(Fold Change) = 0.5, adjusted p-value of < 0.05, medium confidence interaction score (0.4), and FDR ≤ 0.05* (34)*.*


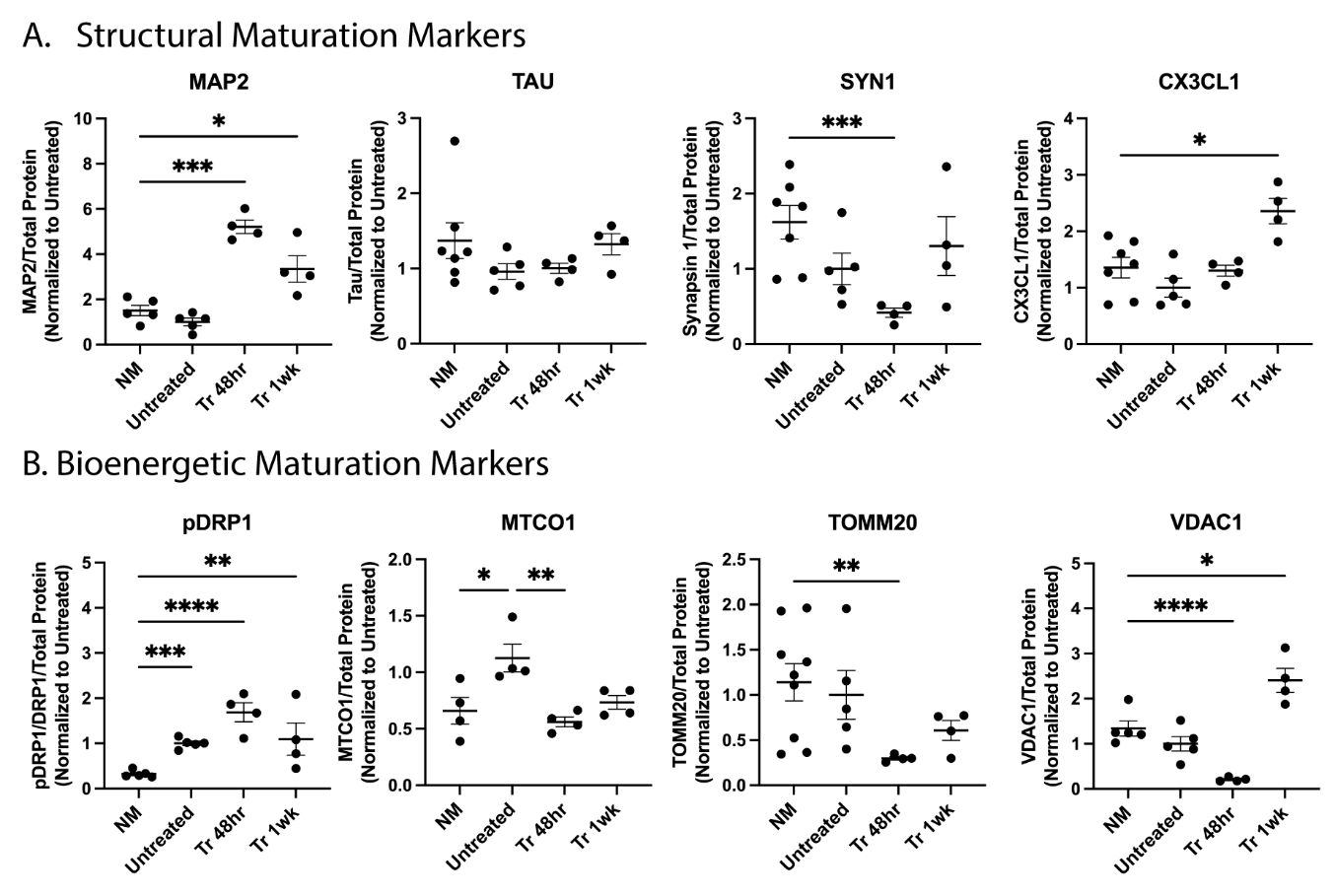


# *Figure S5: Media Treatment – Structural and Bioenergetic Maturation Markers:* ***A.*** *Structural maturation markers (MAP2, Tau, Synapsin-1, and CX3CL1) in NM, Untreated, 48hr Media Treated, and 1wk Media Treated neurons.* ***B.*** *Bioenergetic maturation markers (pDRP1/DRP1, MTCO1, TOMM20, and VDAC1). Western blot quantification was normalized to total protein per lane and expressed relative to the Untreated condition. n = 6 for NM and Untreated; n = 4 for 48hr Treated; n = 4 for 1wk Treated. Mean ± SEM. One-way ANOVA with Tukey post-hoc test, α = 0.05. ROUT outlier analysis applied using GraphPad Prism.*


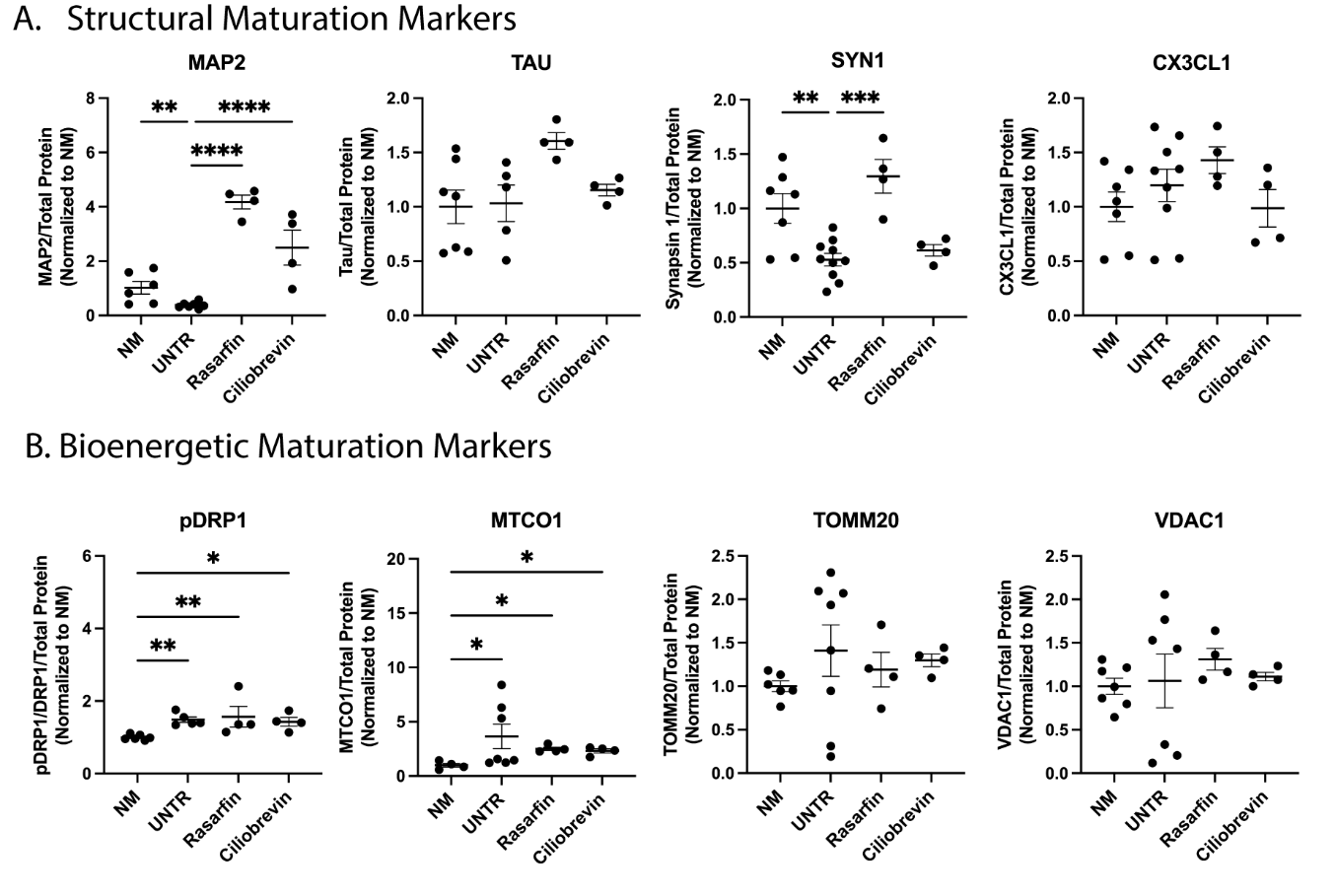


*Figure S6: Mitochondrial Transfer Inhibition – Structural and Bioenergetic Maturation Markers:* ***A.*** *Structural maturation markers (MAP2, Tau, Synapsin-1, and CX3CL1) quantified in NM, Untreated, and NM cultures treated with mitochondrial-transfer inhibitors (10 mM Rasarfin and 10 mM Ciliobrevin).* ***B.*** *Bioenergetic maturation markers (pDRP1/DRP1, MTCO1, TOMM20, and VDAC1). Western blot quantification was normalized to total protein concentration per lane and expressed relative to the Untreated condition. n = 6 for NM and Untreated; n = 4 for Rasarfin Multiple Dose; n = 4 for Ciliobrevin Multiple Dose. Mean ± SEM. One-way ANOVA with Tukey post-hoc test, α = 0.05. ROUT outlier analysis applied using GraphPad Prism.*

*
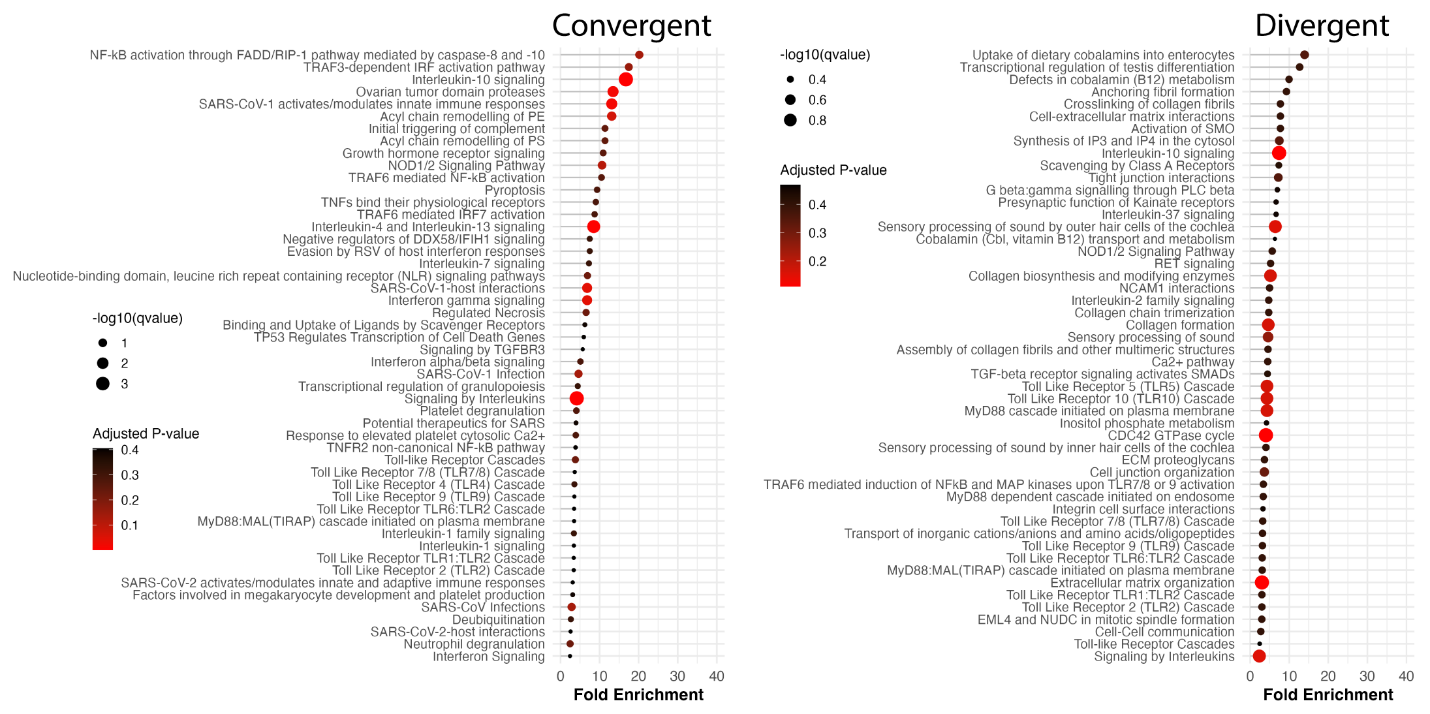
*

*Figure S7: Pathway enrichment analysis between neurons treated with microglial mitochondria and untreated neurons against ASD-associated pathways. The lollipop plot demonstrates pathways significantly enriched as convergent (left) or divergent (right) between NTr and untreated conditions. Convergent pathways include immune and inflammatory signaling modules such as NF-κB activation through FADD/RIP1, TNF receptor signaling, TRAF6-mediated cascades, TLR4 and IL-1 signaling, and nucleotide-binding domain receptor pathways. Divergent pathways include extracellular-matrix and metabolic programs such as collagen biosynthesis and modifying enzymes, glycosaminoglycan metabolism, ECM proteoglycans, dietary cation/anion transport, as well as sensory and GPCR-linked processes (olfactory and taste receptor pathways, ERK/MAPK signaling). Circle size corresponds to –log10(adjusted p-value), and color represents the adjusted p-value.*

*
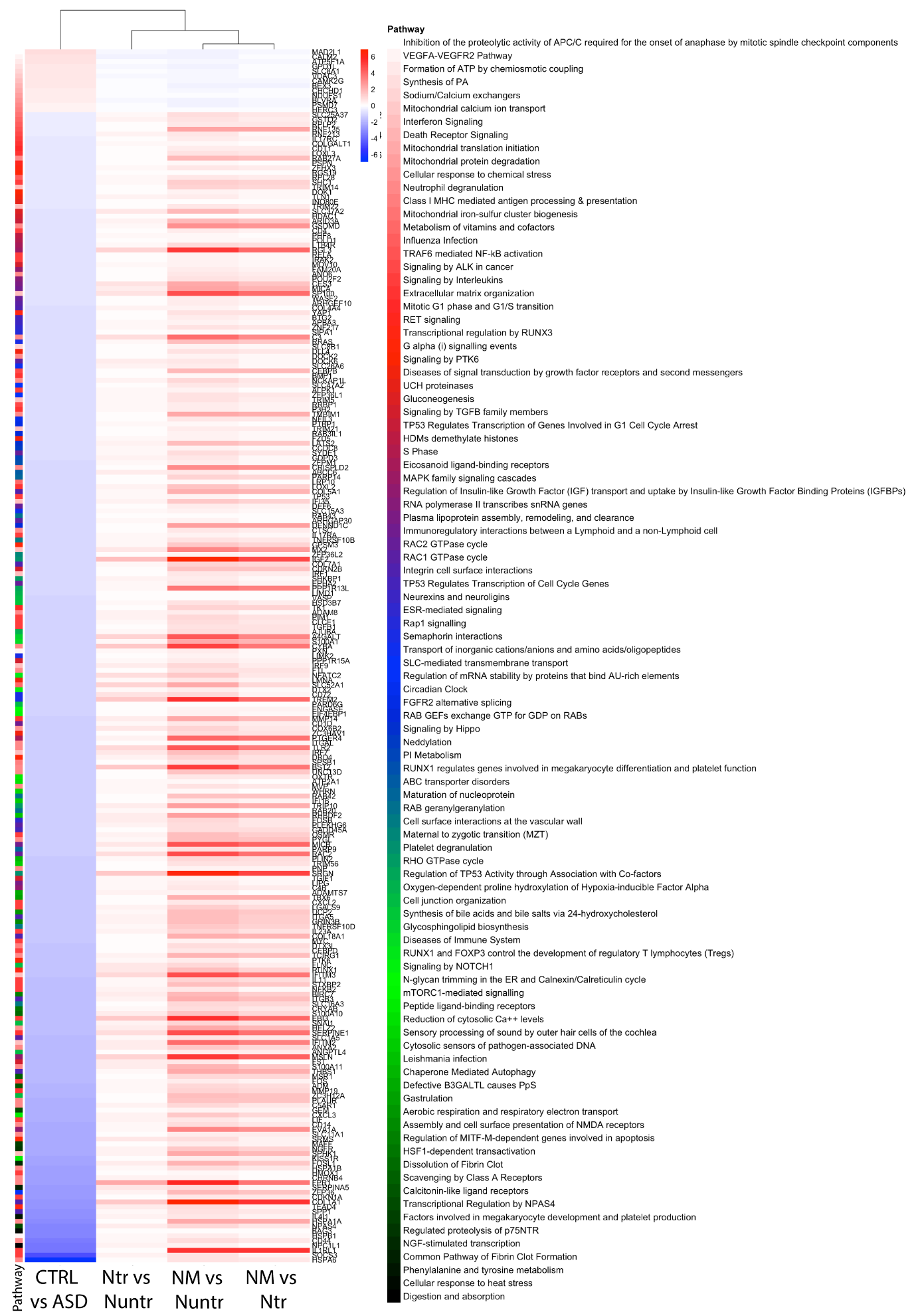
*

*Figure S8:* ***Divergent pathway analysis shows that both conditions move away from ASD signatures on synaptic, chromatin, and mitochondrial programs.****Heatmap of pathway enrichment comparing the cortex of ASD patients to neuronal cultures treated for 48hr with microglial mitochondria (NTr), neuron–microglia co-cultures (NM), and untreated neuron monocultures (CTRL). Pathway enrichment analysis between ASD patients, mitochondria-treated, and NM groups. Log_2_(Fold Change) = 0.2, adjusted p-value of < 0.05, medium confidence interaction score (0.4), and FDR ≤ 0.05* [34]*.*
